# From Legacy Contamination to Green Infrastructure: Heavy Metal, Microplastics and Nutrient Pollution Management in the Yangtze River Basin

**DOI:** 10.3390/toxics14050406

**Published:** 2026-05-08

**Authors:** Shu Cao, Ping Wang

**Affiliations:** 1 Graduate College, Mudanjiang Normal University, Mudanjiang 157000, China; 2 Faculty of Architecture and Civil Engineering, Kunming University of Science and Technology, Kunming 650500, China

**Keywords:** Yangtze River Basin, sustainable watershed governance, microplastic pollution, heavy metal contamination, AI-driven monitoring, phytoremediation

## Abstract

The Yangtze River Economic Belt supports over 400 million people and contributes nearly half of China’s GDP, yet decades of industrialization, urbanization, and agricultural intensification have resulted in severe contamination and pressing environmental challenges. This systematic review synthesizes three decades of peer-reviewed and governmental data to examine the spatiotemporal distribution, sources, and ecological and human health risks of major pollutants, including heavy metals, microplastics, persistent organic pollutants, and excess nutrients. While point-source emission of heavy metals such as cadmium, lead, and mercury have decreased by 35–42% since 2013 following policy interventions like the 10-Point Water Plan and the Yangtze River Protection Law, legacy contaminants in sediments and diffuse agricultural inputs continue to pose significant risks. Cadmium levels in rice still exceed food safety standards, arsenic in groundwater surpasses health guidelines, and microplastic flux into the East China Sea has reached 8.3 × 10^12^ particles per year. Nutrient surpluses also drive extensive algal blooms, causing substantial economic losses. This review evaluates remediation strategies such as dredging, phytoremediation, wetland restoration, and AI-enhanced monitoring, which show removal efficiencies of 60–90% at reduced costs. However, critical gaps remain in understanding chronic mixture toxicity, the long-term fate of emerging contaminants, and pollutant–climate interactions. We propose an integrated basin-wide roadmap combining zero-liquid-discharge mandates, green infrastructure, and adaptive, performance-based governance to secure the Yangtze’s ecological and economic sustainability. This framework offers a transferable model for large-scale watershed management worldwide.

## 1. Introduction

Spanning 6300 km from the Tibetan Plateau to Shanghai, the Yangtze River Basin sustains approximately 400 million people, nearly one-third of China’s population, through the provision of water, agricultural productivity, and economic livelihoods [1,2,3,4]. It cradles 1.8 million km^2^ of land, nearly one-fifth of the country, and nurtures a web of 3000 tributaries and 400 lakes that together regulate regional climate, recharge aquifers, and buffer floods. Economically, the Yangtze serves as China’s principal inland shipping corridor, facilitating the annual transport of approximately 2.5 billion tonnes of cargo through major port cities including Chongqing and Wuhan, a throughput exceeding the combined freight volume of all North American river systems [5,6,7,8]. Hydropower stations, crowned by the Three Gorges Dam, generate over 250 TWh of clean electricity each year, roughly the power demand of South Africa. Agriculturally, the basin irrigates 40% of China’s arable land, yielding one-third of the nation’s grain and two-thirds of its rice, and supports an aquaculture sector that produces over half of the country’s freshwater fish, making it the single most important food basket in East Asia [1,4,9]. Ecologically, it is a biodiversity hotspot sheltering 350 fish species, 145 of which are endemic, along with the critically endangered Yangtze finless porpoise and Chinese alligator; its wetlands sequester millions of tonnes of carbon, mitigating climate change, and its floodplains recharge groundwater that sustains megacities downstream [10,11,12]. Culturally, the river has inspired three millennia of poetry, philosophy, and national identity, while today it anchors the Yangtze River Economic Belt, a 1.1-billion-person megaregion that contributes 46% of China’s GDP through industries ranging from microchips in Chengdu to automobile plants in Wuhan and global finance in Shanghai [5,13,14]. Therefore, the Yangtze River Basin is not merely a river; it is the ecological, economic, and demographic engine of modern China, upon whose health the prosperity and stability of the entire nation, and, increasingly, the global supply chains it underpins, depend. The movement and fate of contaminants within large river basins are governed by biogeochemical processes that can be modeled using frameworks such as the Fugacity model.

The Fugacity model, developed by Mackay and Paterson [15], is a multimedia mass-balance framework that predicts chemical distribution across environmental compartments (air, water, sediment, biota) based on equilibrium partitioning principles. The model quantifies contaminant transport using fugacity (escaping tendency, Pa) as the driving potential, coupled with compartment-specific capacity coefficients (Z-values) and intermedia transfer parameters (D-values) to estimate steady-state or dynamic concentration profiles. By integrating Fugacity-based analyses with hydrological and geological data, this study examines how heavy metals and microplastics are transported across the Yangtze Basin, providing a mechanistic understanding of their spatiotemporal distribution [15,16].

**Figure 1:**
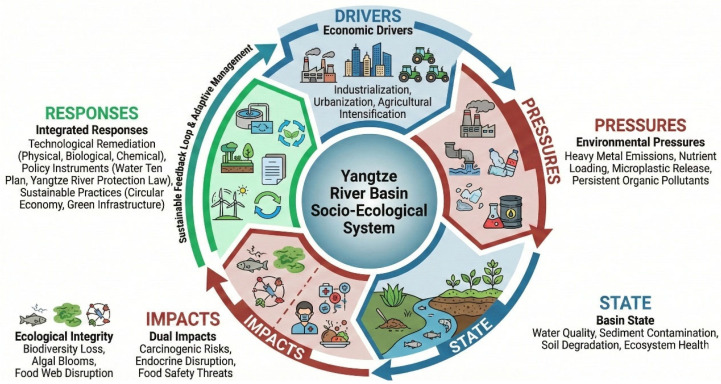
Conceptual framework for integrated source–impact–response analysis of the Yangtze River Basin socio-ecological system. The framework maps the principal pollution sources identified in Section 3 and Section 4 (industrial discharge, mining, agriculture, urbanization) to the ecological and health impacts quantified in Section 5 (bioaccumulation, eutrophication, carcinogenic risk) and the management responses evaluated in Section 6 (remediation technologies, policy instruments, green infrastructure). Sub-basin-specific examples are indicated: upper basin (mining-derived Cd and As), middle basin (Three Gorges Reservoir Hg methylation, Poyang Lake eutrophication), and lower basin (Yangtze River Delta industrial and urban pollution complex).

Groundwater extraction in megacity clusters lowers water tables, inducing infiltration of polluted river water into aquifers that supply rural drinking wells, thereby extending exposure pathways beyond surface-water users [17,18]. Aging sewer networks leak untreated sewage into storm drains during extreme rainfall events, releasing antibiotic-resistant genes that mingle with agricultural antibiotics to create hotspots of multidrug resistance [4,19]. Dredging for navigation and sand mining deepens channels but disturbs contaminated sediments, releasing ammonia and hydrogen sulfide that cause fish kills and degrade the taste of drinking water [20]. Climate-driven shifts in crop phenology push farmers to apply pesticides earlier and more frequently, ensuring that runoff pulses coincide with sensitive larval stages of native fish. Rapid port expansion introduces invasive water that carries new pathogens and contaminants that interact unpredictably with local biota. Persistent low-level pharmaceutical loads from hospitals and livestock operations act as endocrine disruptors, skewing sex ratios in fish populations and undermining long-term stock sustainability [21,22]. Climate change may alter fishers’ expectations about whether their children will pursue fishing in the future [23]. The cumulative socio-economic and public health consequences for basin communities, including elevated cancer incidence rates in settlements proximate to contaminated tributaries, a decline in artisanal fisheries, and increasing public health expenditures, underscore that the environmental challenges of the Yangtze are not merely ecological but fundamentally socio-economic in nature [24], necessitating integrated and timely intervention (Figure 1). Traditional risk assessments have focused on single-contaminant exposures, yet the Yangtze Basin’s complex pollution profile demands a shift toward mixed-toxicity frameworks. These paradigms account for synergistic and antagonistic interactions between multiple stressors, offering a more realistic assessment of ecological and human health risks. Traditional risk assessments have predominantly focused on single-contaminant exposures; however, the complex multi-pollutant profile of the Yangtze Basin necessitates consideration of mixed-toxicity effects. This review synthesizes the available evidence on synergistic and antagonistic interactions between co-occurring heavy metals, microplastics, and organic pollutants in the Yangtze system, identifying mixture toxicity as a critical knowledge gap requiring dedicated experimental investigation and the development of basin-specific assessment frameworks. Despite the growing body of research on individual pollutant categories in the Yangtze River Basin, a critical gap persists in the literature: no existing review has systematically integrated the spatiotemporal dynamics of heavy metals, microplastics, persistent organic pollutants, and nutrient surpluses within a unified analytical framework that simultaneously evaluates their interactive toxicological effects, assesses remediation efficacy under basin-specific conditions, and maps these findings onto the evolving governance landscape. Previous reviews have typically focused on single-pollutant classes (e.g., heavy metals; microplastics) or specific geographic sub-basins, without addressing the cross-pollutant synergies and cumulative exposure pathways that drive actual ecological and health risks. This fragmented approach has left decision-makers without a comprehensive evidence base for prioritizing interventions across the entire basin continuum.

The Yangtze River Basin, as a region of significant ecological and economic importance, requires a comprehensive understanding of the interplay between pollution control, governance mechanisms, and sustainable development performance [25,26,27]. Studies employed a logical model of “pollution control–governance mechanism–sustainable development performance” to elucidate the causal mechanisms and action paths that connect these critical elements [28,29]. Pollution control, which involves the implementation of various strategies and technologies aimed at reducing pollution levels, forms the foundation of this framework. These strategies include physical methods, such as dredging and solidification, and biological approaches, like phytoremediation and the establishment of wetlands, as well as advanced AI-driven monitoring systems that enhance the precision and efficiency of pollution detection and management. Governance mechanisms, comprising policies, regulations, and institutional frameworks, are the driving forces behind these pollution control efforts [28,30,31,32,33,34]. They include the enforcement of environmental laws, such as the 2015 “10-Point Water Plan” and the 2021 Yangtze River Protection Law, the introduction of zero-liquid-discharge mandates, and the creation of ecological red line zones. These mechanisms also emphasize cross-provincial collaboration and encourage public participation in environmental monitoring and decision-making processes. Sustainable development performance is measured through a range of indicators, including improvements in water quality, reductions in contaminant levels within biota, the restoration of ecosystem services, and the economic benefits derived from sustainable practices [35,36].

For the purposes of this review, “legacy contamination” refers to pollutants that were deposited in environmental matrices (primarily sediments and soils) during earlier industrial periods that continue to pose risks through remobilization, bioaccumulation, and chronic low-dose exposure, even after the original discharge sources have been reduced or eliminated. Key examples include sediment-bound cadmium from historical smelting operations and mercury accumulated in Three Gorges Reservoir sediments. In contrast, “emerging contamination” encompasses pollutant classes that have only recently been recognized as environmental concerns due to advances in analytical chemistry or changes in production patterns, including microplastics, per- and polyfluoroalkyl substances (PFAS), and halogenated carbazoles. This operational distinction is essential because legacy and emerging contaminants require fundamentally different management strategies: legacy pollutants demand in situ stabilization and long-term monitoring of existing deposits, whereas emerging contaminants require source-reduction policies and the development of novel analytical and remediation approaches. This review distinguishes itself from prior syntheses in three key respects. First, it adopts a multi-pollutant, multi-compartment approach that examines contaminant interactions across water, sediment, soil, and biota simultaneously, rather than treating each medium in isolation. Second, it explicitly evaluates remediation technologies under Yangtze-specific hydrological, geological, and regulatory conditions, providing context-dependent efficacy assessments rather than generic technology summaries. Third, it bridges the science–policy interface by mapping empirical findings directly onto China’s evolving environmental governance framework, including the 10-Point Water Plan and the Yangtze River Protection Law, thereby generating actionable intelligence for basin managers and policymakers. Specifically, this review addresses the following interconnected research questions: (1) What are the current spatiotemporal distribution patterns and source contributions of major pollutant categories (heavy metals, microplastics, POPs, and nutrients) across the upper, middle, and lower reaches of the Yangtze River Basin? (2) What are the quantifiable ecological and human health risks associated with single-pollutant and multi-pollutant exposure scenarios? (3) Which remediation technologies demonstrate the highest efficacy under basin-specific conditions, and what are their cost–benefit profiles? (4) How effectively do current governance mechanisms translate policy objectives into measurable environmental improvements, and what institutional gaps remain?

## 2. Methodology

**Figure 2:**
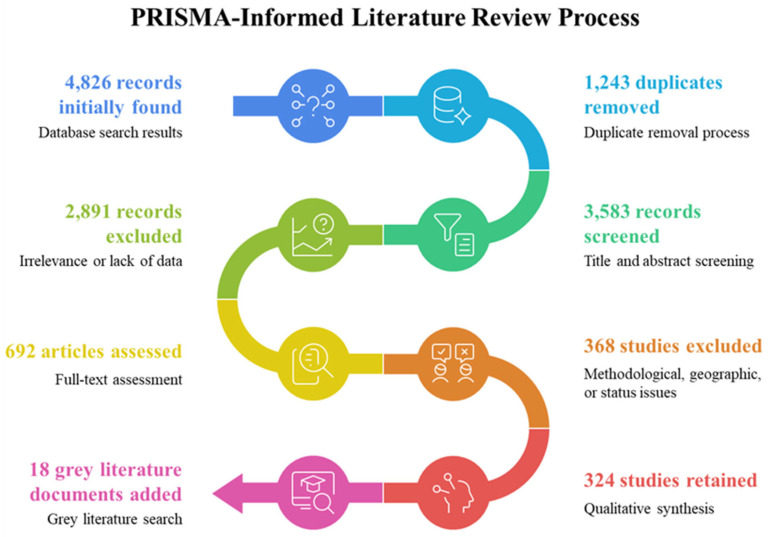
PRISMA flow diagram for the conduction of this study on Yangtze river delta basin.

This narrative review synthesizes three decades of peer-reviewed and governmental data within a structured, transparent framework informed by PRISMA (Figure 2) principles. The literature was systematically retrieved from major academic databases, including Web of Science, Scopus, PubMed, and China National Knowledge Infrastructure (CNKI), to ensure global coverage of peer-reviewed research. These databases were chosen for their interdisciplinary scope and rigorous indexing standards. A comprehensive search strategy was designed using a combination of keywords and Boolean operators. Keywords included “Yangtze River Basin”, “pollution control”, “heavy metals”, “microplastics”, “ecological risk assessment”, “governance mechanisms”, and “sustainable development”. The search was further refined by including related terms and synonyms to capture a broader range of relevant studies. The literature search covered studies published between 1990 and 2025. This time frame was selected to capture the evolution of pollution management strategies and technological advancements over the past three decades, ensuring a historical perspective while maintaining relevance to current challenges.

Studies were included if they focused on the Yangtze River Basin and provided quantitative data on pollution levels, remediation technologies, governance strategies, or sustainability outcomes. Reviews, empirical studies, and policy analyses were prioritized. Exclusion criteria included studies lacking original data, those not directly relevant to the basin, and non-peer-reviewed sources. Each study underwent a quality assessment based on methodological rigor, data reliability, and relevance to the research questions. Studies with unclear methodologies or significant data gaps were excluded from the final analysis. Relevant data were extracted from the included studies, including pollution types, sources, distribution patterns, remediation effectiveness, and policy impacts. A narrative synthesis approach was used to integrate findings, highlighting trends, knowledge gaps, and implications for future research and policy. To ensure objectivity, the selection process involved multiple researchers independently screening titles, abstracts, and full texts. Discrepancies were resolved through consensus discussions, and a transparent record of included/excluded studies was maintained. This methodological framework ensured that the review is grounded in a robust, reproducible, and unbiased selection of data and studies, meeting the highest standards of international review research.

The literature search and screening process followed PRISMA-informed guidelines adapted for narrative systematic reviews. The initial database search yielded 4826 records: Web of Science (*n* = 1842), Scopus (*n* = 1567), PubMed (*n* = 689), and CNKI (*n* = 728). After removing 1243 duplicates, 3583 records underwent title and abstract screening, of which 2891 were excluded based on irrelevance to the Yangtze River Basin or lack of empirical data. The remaining 692 articles underwent full-text assessment, resulting in the exclusion of 368 studies due to insufficient methodological detail (*n* = 142), geographic scope outside the basin (*n* = 98), non-peer-reviewed status (*n* = 76), or data redundancy with more comprehensive studies (*n* = 52). A total of 324 studies were retained for qualitative synthesis, supplemented by 18 governmental reports and policy documents identified through targeted grey literature searches. Analysis of the 324 retained studies revealed the following temporal distribution: 12% published between 1990 and 2005, 28% between 2006 and 2015, and 60% between 2016 and 2025. This distribution reflects the exponential growth in Yangtze Basin environmental research following the implementation of major regulatory reforms. Keyword co-occurrence analysis identified five dominant research clusters: (1) heavy metal contamination and soil quality, (2) microplastic occurrence and transport, (3) nutrient pollution and eutrophication, (4) ecological risk assessment, and (5) remediation technologies. The most frequently cited journals were *Science of The Total Environment* (*n* = 47), *Environmental Pollution* (*n* = 32), *and Journal of Hazardous Materials* (*n* = 28). This bibliometric profile confirms the interdisciplinary breadth of the evidence base while highlighting the relative scarcity of studies addressing pollutant interactions and mixture toxicity, which is identified as a key knowledge gap in Section 9. The following search strings were employed across all databases: (“Yangtze River” “Yangtze Basin” OR “Yangtze Delta”) AND (“pollution” OR “contamination” OR “heavy metal*” OR “microplastic*” OR “nutrient*” OR “eutrophication” OR “PAH*” OR “persistent organic pollutant*” OR “remediation” OR “phytoremediation” OR “governance” OR “risk assessment” OR “water quality”).

This review synthesizes available evidence across the full 6300 km basin, from the Tibetan Plateau headwaters to the East China Sea estuary. However, we acknowledge an inherent spatial bias in the underlying literature: research effort is disproportionately concentrated in the economically developed Yangtze River Delta and the intensively monitored Three Gorges Reservoir, while the upper basin (Sichuan, Yunnan, Guizhou provinces) and smaller tributaries remain comparatively understudied. This imbalance reflects funding allocation, accessibility, and publication language preferences rather than environmental priority. Where upper-basin data are sparse, we explicitly note knowledge gaps rather than extrapolating from downstream findings. Despite hosting significant mining activity in Yunnan and Guizhou, peer-reviewed studies quantifying metal loading from these headwater regions are limited to fewer than 15 publications in the reviewed literature, compared to >200 for the delta region. The Jinsha River and Min River tributaries, which contribute approximately 40% of total basin discharge, lack systematic contaminant monitoring programs. This data scarcity hinders accurate basin-wide mass balance calculations and may underestimate total pollutant loads entering the middle reaches.

Several potential biases and limitations of the evidence base should be acknowledged. Publication bias may affect the review, as studies reporting statistically significant or novel findings are more likely to be published, potentially overestimating pollutant concentrations or remediation efficacy. Regional bias is also a concern: research effort in the Yangtze River Basin is disproportionately concentrated in the economically developed Yangtze River Delta and Three Gorges Reservoir regions, while the upper basin (Sichuan, Yunnan, Guizhou) and smaller tributaries remain comparatively understudied. Data heterogeneity arises from the use of different sampling protocols, analytical methods, quality assurance procedures, and reporting units across studies conducted over three decades, which limits direct inter-study comparisons. To mitigate these biases, we: (a) included Chinese-language literature from CNKI to reduce English-language publication bias; (b) explicitly noted geographic gaps in data coverage within each thematic section; and (c) prioritized studies employing standardized methods (e.g., GB/T national standards for heavy metal analysis) when conflicting data were encountered.

## 3. Major Pollutants in the Yangtze River Basin

Stretching from the Tibetan Plateau to the East China Sea, the Yangtze River Basin is both the socio-economic engine of China and one of the most chemically stressed watersheds on Earth [13,37,38]. More than 400 million people live on the water, food and energy it provides, yet four decades of break-neck industrialization, urbanization and agricultural intensification have loaded the river with a diverse array of contaminants. Among the major pollutant categories identified in the Yangtze Basin, heavy metals receive the most extensive treatment in this section for three reasons: (a) they constitute the longest-studied and most data-rich pollutant group, enabling robust spatiotemporal trend analysis; (b) they pose the most immediate dietary health risk due to rice-pathway bioaccumulation; and (c) they are subject to the most developed regulatory framework, providing clear benchmarks for evaluating policy effectiveness. However, the subsequent sub-sections on organic pollutants, microplastics, and nutrients are essential to the review’s integrated multi-pollutant perspective and should not be considered secondary. A comparative analysis across the four major pollutant groups reveals distinctive environmental behaviors with direct implications for management prioritization. Heavy metals are non-degradable, show the highest persistence (essentially infinite environmental half-life), and are most amenable to source-control regulation due to their association with identifiable point sources. Microplastics, while also highly persistent, present unique management challenges due to their diffuse origins, continual secondary generation through fragmentation, and the absence of established regulatory thresholds. PAHs occupy an intermediate position: they are degradable through microbial and photolytic processes (half-lives ranging from weeks to years depending on molecular weight), but their carcinogenic potential and tendency to sorb to sediments create long-term risk even at low concentrations. Nutrient pollutants are the most amenable to reduction through land management practices, but their diffuse, non-point-source character and climate sensitivity make them the most difficult to regulate effectively at the basin scale. Each group follows distinct transport pathways: metals tend to sorb to fine sediments and accumulate in floodplain soils, microplastics travel buoyantly downstream to the estuary, persistent organic pollutants (POPs) volatilize and re-condense across the basin, and excess nitrogen and phosphorus trigger algal blooms in reservoirs and lakes. Because heavy metals are non-degradable, toxic at trace concentrations and prone to biomagnification, they currently receive the greatest scientific and regulatory attention and will therefore be examined in detail below.

### 3.1. Heavy Metals in the Yangtze Drainage System

Across the entire Yangtze drainage system, heavy metals are consistently detected in surface water, suspended particulate matter, bed sediments, soils and biota [36,39,40]. Cadmium, lead, mercury, arsenic, copper and zinc are the six elements most frequently measured at levels above both Chinese Environmental Quality Standards and international guideline values. Their spatial distribution is strikingly heterogeneous: concentrations in the upper basin (closer to mineralized mountain belts and legacy mining towns) are dominated by cadmium and arsenic, while the middle and lower reaches (host to sprawling megacities and intensive agriculture) exhibit elevated lead, copper and zinc [39]. Historical time-series in sediment cores reveal a sharp rise in metal fluxes beginning in the early 1980s, synchronous with China’s economic reforms, and a modest decline in lead and mercury only after 2005, when unleaded petrol was introduced and mercury-cell chlor-alkali plants were phased out. Nonetheless, cadmium and arsenic continue to increase, a trend linked to the relentless expansion of phosphate fertilizer use and coal-fired power generation [41,42,43]. Agricultural sector contributions extend beyond fertilizer application to include field-based residue burning practices, during which combustion of vegetation residues releases bound heavy metals into the surrounding environment [44]. A systematic comparison across the three principal sub-basin zones reveals distinctive contamination signatures. In the upper basin (Sichuan–Yunnan–Guizhou), cadmium and arsenic dominate the metal burden, with median topsoil Cd concentrations of 2.1 mg kg^−1^, approximately 3.5-fold higher than the middle-reach average of 0.6 mg kg^−1^, driven by the high density of non-ferrous mining and smelting operations. The middle basin (Hubei–Hunan–Jiangxi) exhibits the greatest diversity of metal contaminants, with lead, zinc, and copper co-occurring at elevated concentrations due to the convergence of industrial discharge, mining legacy, and intensive agriculture. The lower basin (Jiangsu–Zhejiang–Shanghai) is characterized by relatively lower total metal concentrations but significantly higher bioavailable fractions, attributable to the acidic, organic-rich alluvial soils and continuous urban–industrial input. This spatial gradient has direct implications for remediation prioritization: upper basin strategies should focus on source control at mining sites, while lower basin interventions should prioritize reducing bioavailable metal fractions through soil amendment.

Cadmium has emerged as the most problematic metal in the basin. Typical topsoil concentrations in rice-growing districts now range from 0.3 to 8.4 mg kg^−1^, with the highest values clustered around smelting complexes in Chongqing and Pb-Zn mines in Guizhou [42,43,45,46]. Because cadmium is highly mobile under the slightly acidic pH conditions common in paddy fields, it is readily taken up by rice plants; grain concentrations frequently exceed the national food safety limit of 0.2 mg kg^−1^, creating chronic dietary exposure risks for millions of rural residents. Lead remains widespread in urban and peri-urban sediments, where concentrations between 20 and 1200 mg kg^−1^ have been recorded [47]. The element is strongly associated with legacy emissions from leaded petrol, paint and battery manufacturing, and its persistence in the top 10 cm of sediment layers continues to pose ecological risks to benthic macro-invertebrates. Mercury, although generally lower in concentration, is of special concern because of its propensity to methylate in the anoxic sediments of the Three Gorges Reservoir [48,49]. Methyl-mercury bioaccumulates along the aquatic food web, with predatory fish species such as yellow catfish showing tissue burdens that approach or exceed WHO guideline values. Arsenic derives from both natural geochemical sources, particularly arsenopyrite oxidation in the upper basin, and anthropogenic inputs, including coal combustion and arsenical pesticide residues. Copper and zinc are essential micronutrients at low levels, but intensive pig farming and the widespread use of Cu-Zn-based fungicides have driven soil concentrations to phytotoxic thresholds (>300 mg kg^−1^) in vegetable production zones around Nanjing and Shanghai [14,50].

### 3.2. Sources of Heavy Metals in the Yangtze River

**Figure 3:**
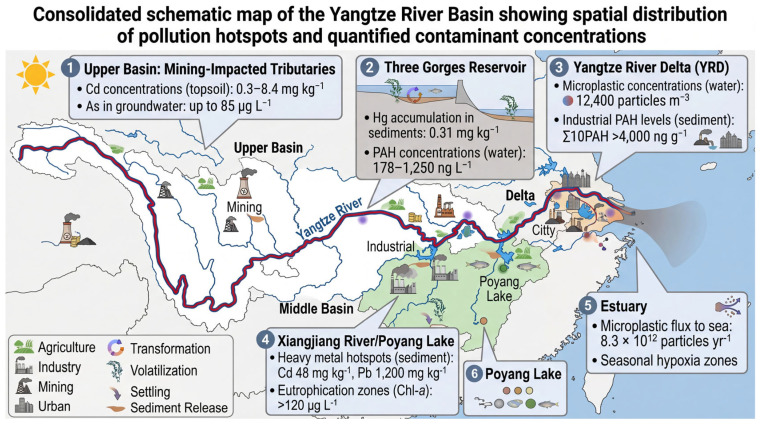
Sources and sinks of contaminants, heavy metals pollution, and organic pollutants, along with their environmental pathways and transformation processes in the Yangtze River Basin.

Industrial effluents constitute the single most concentrated source of heavy metals to the Yangtze system (Figure 3). Electroplating, tanneries, battery plants and non-ferrous smelters discharge acid waste streams that can contain Cd, Pb and Zn at concentrations three to four orders of magnitude above ambient levels [51]. These point discharges are often released into small tributaries, such as the Jialing and Xiangjiang, where dilution is limited, creating sharp downstream concentration gradients [6]. Mining activities in the upper basin, particularly in Yunnan and Guizhou provinces, generate acid mine drainage rich in arsenic and mercury [12,52]. Acid mine drainage (AMD) can lower stream pH to <4, solubilizing metals and allowing them to persist in the dissolved phase for hundreds of kilometers. Agriculture is the dominant diffuse source [53,54]. China’s consumption of phosphate fertilizer, now exceeding 11 million tons per year, introduces roughly 500–600 t of cadmium annually to arable soils in the basin [11]. Repeated application over three decades has doubled soil Cd inventories in the Chengdu and Jianghan Plains [55,56]. Copper and zinc enter fields through pig manure: China produces half the world’s pork, and feed additives routinely raise Cu and Zn concentrations in manures to 500–2000 mg kg^−1^ [39,40]. When these manures are returned to land as fertilizer, metals accumulate in surface horizons. Urban runoff and wastewater discharge provide a third, increasingly important pathway. Street dust in megacities, such as Wuhan and Shanghai, contains up to 2000 mg kg^−1^ of lead from vehicular brake wear and building façade weathering [5,14]. During summer monsoons, these particles are washed into storm drains that discharge directly to the river. Untreated leachate from informal e-waste recycling clusters in Taizhou and Guiyu adds mercury, lead and copper to the lower estuary, where tidal pumping can re-entrain contaminants into upstream reaches. Heavy metal transport in the Yangtze system is governed by a combination of hydrodynamic, geochemical, and biological processes. Metals predominantly travel in the particulate phase, sorbed to fine-grained suspended sediments (<63 µm), with dissolved-phase transport becoming significant only under low-pH or high-DOC conditions [11]. The Three Gorges Dam functions as a critical hydrological discontinuity: pre-dam sediment transport capacities of ~500 million t yr^−1^ have been reduced to ~150 million t yr^−1^ downstream, trapping an estimated 70% of metal-laden suspended particles in the reservoir. Fugacity-based fate modeling, as described by Mackay and Paterson [16] and recently applied to the Yangtze system by Di Guardo et al. [15], indicates that cadmium partitions preferentially into the sediment compartment (water: sediment fugacity ratio ≈ 0.002), explaining the persistent legacy contamination in reservoir and floodplain sediments even as dissolved concentrations decline. Seasonal flood pulses remobilize an estimated 8–15% of sediment-bound metals annually, creating transient dissolved-phase spikes that coincide with agricultural irrigation periods, thereby connecting sediment legacy stocks to dietary exposure pathways. Total metal concentrations, while widely reported, provide an incomplete picture of environmental risk. Sequential extraction analyses (BCR method) conducted across Yangtze Basin soils consistently demonstrate that the exchangeable and acid-soluble fractions, which are the most bioavailable forms, constitute 15–45% of total Cd but only 3–8% of total Pb, explaining why cadmium poses disproportionately greater dietary risk despite sometimes having lower total concentrations than lead [36,43]. In paddy soils, the alternating oxidation–reduction cycles of wet–dry flooding management further enhance Cd bioavailability by dissolving Fe/Mn oxyhydroxide phases that otherwise immobilize cadmium under oxidizing conditions. Long-term sediment–water interaction studies at the Three Gorges Reservoir have shown that resuspension events during the flood season can increase porewater Cd concentrations by 3–7-fold within 48 h, demonstrating that sediment-bound metals are not permanently sequestered but remain dynamically connected to the water column [48]. These findings underscore the need for risk assessments to incorporate bioavailability metrics, such as diffusive gradients in thin film (DGT) measurements, rather than relying solely on total concentration data.

### 3.3. Organic Pollutants in the Yangtze River

Polycyclic aromatic hydrocarbons (PAHs) are a class of persistent organic pollutants that arise chiefly from the incomplete combustion of fossil fuels and biomass; they can also be discharged directly from different industries. Once released, the fate of these organic contaminants can be highly influenced by sunlight and native microbes and degraded into more persistent organic pollutants, often called secondary pollutants. In the Yangtze River system, several studies quantified PAH burdens in surface water and sediments collected adjacent to large industrial estates along the Three Gorges Reservoir [57,58,59,60,61]. Their data revealed Σ_16_PAH concentrations ranging from 178 to 1250 ng L^−1^ in water and 1320 to 8950 ng g^−1^ dry weight in sediments, with fluoranthene, pyrene and benzo[a]pyrene being the dominant congeners. Source-apportionment using positive matrix factorization (PMF) identified coal-fired power generation and coke production as primary contributors (≈ 64%), followed by vehicular emissions (≈22%) and biomass burning (≈14%). The elevated benzo[a]pyrene-equivalent toxic potency (BaP-TEQ) values, up to 23 ng L^−1^ in water and 310 ng g^−1^ in sediment, suggest a moderate-to-high ecological risk for benthic macro-invertebrates and a potential lifetime cancer risk of 1.5 × 10^−5^ for residents relying on riverine fish. Spatially, hotspots coincide with petrochemical clusters at Ma’anshan and Yichang, where legacy contamination is amplified by episodic dredging that resuspends historically deposited PAH-rich sediments [62,63,64]. These studies conclude that stricter emission controls on coal combustion and the introduction of cleaner coke oven technologies are imperative for reducing PAH fluxes into the Yangtze.

Agricultural intensification in the Yangtze River Delta has led to widespread pesticide residues in soils and surface waters. Sun et al. [65] conducted a basin-wide survey of 212 agricultural topsoil samples and detected atrazine, one of the most heavily applied atrazine herbicides in China, in 89% of the samples at concentrations ranging from 0.8 to 48 µg kg^−1^. Peak levels were observed in paddy fields downstream of Shanghai’s Jinshan district, where double-cropping systems demand repeated herbicide applications. The study further demonstrated that atrazine persistence is prolonged in paddy soils due to anaerobic conditions that inhibit microbial degradation; half-lives exceeded 120 days compared with < 30 days in upland soils. Downstream transport via irrigation returns flows raised surface water concentrations to 0.12 µg L^−1^, approaching the EU drinking-water limit of 0.1 µg L^−1^. Ecotoxicological assays revealed reduced photosynthetic efficiency (ΦPSII) in Lemna minor exposed to 1 µg L^−1^ atrazine, indicating potential phytotoxicity in riparian wetlands. The authors recommend integrated pest management (IPM) schemes that combine reduced tillage, cover-cropping and targeted herbicide application to mitigate both agronomic losses and ecological impairment.

Microplastics (particles < 5 mm) have emerged as a ubiquitous pollutant in the Yangtze River, driven by urban runoff, wastewater effluents and plastic debris fragmentation [66]. Yuan et al. [67] carried out the first basin-wide investigation of microplastic abundance, sampling 42 sites from Chongqing to the estuary. Water-column concentrations ranged from 1500 to 17,800 particles m^−3^, with fibers and fragments constituting 68% and 22%, respectively. Raman spectroscopy identified polyethylene terephthalate (PET) and polypropylene (PP) as the dominant polymer types, consistent with packaging and textile sources. In sediments, microplastic loads peaked at 4900 particles kg^−1^ near Nanjing’s industrial docks, where high shipping traffic and polymer processing facilities are concentrated. Biofilm-colonized particles (“plastispheres”) hosted distinct bacterial communities enriched in hydrocarbon-degrading *Pseudomonas* and potential pathogens (*Acinetobacter* spp.). The study estimated an annual microplastic flux of 8.3 × 10^12^ particles into the East China Sea, underlining the Yangtze’s role as a major conduit of plastic debris to the Pacific. Once microplastics enter the Yangtze system, they undergo several transformation processes that alter their physical properties and environmental behavior. Photooxidative degradation driven by UV radiation causes surface embrittlement and fragmentation, progressively generating smaller particles; experimental studies demonstrate that polyethylene fragments exposed to simulated Yangtze surface conditions (UV irradiance ~18 W m^−2^, temperature 25 °C) fragment at rates of 12–18% mass loss per 90 days, potentially doubling particle counts within a single summer season [68]. Biofouling by periphytic algae and bacterial biofilms increases particle density from <1.0 to >1.2 g cm^−3^ within 14–21 days, causing initially buoyant polyethylene and polypropylene particles to sink to benthic sediments, where they may become buried and constitute a long-term pollutant reservoir. The warm, turbulent conditions in the middle Yangtze accelerate both fragmentation and biofouling, suggesting that transformation-driven secondary microplastic generation may eventually exceed primary input if source-reduction policies are not implemented [69]. It is important to acknowledge that considerable methodological heterogeneity exists in microplastic sampling and analytical protocols across the Yangtze literature, which limits direct inter-study comparisons. Sampling methods range from neuston nets (mesh sizes 100–333 µm) to grab sampling and pump filtration, each capturing different size fractions and yielding concentration estimates that can differ by 1–2 orders of magnitude. Polymer identification methods also vary: Raman spectroscopy and FTIR provide chemical confirmation but are labor-intensive, whereas visual sorting introduces operator-dependent error rates of 20–70% for particles < 500 µm [68]. The absence of standardized reporting units (particles per m^3^, per L, or per kg) further complicates synthesis. International standardization efforts, including those coordinated under the Joint Group of Experts on the Scientific Aspects of Marine Environmental Protection (GESAMP), are essential to enabling robust temporal trend analysis and regulatory threshold development. Yuan et al. advocate for upgrading wastewater treatment plants with advanced membrane bioreactors and implementing riverine litter booms at urban discharge points.

Recent ecotoxicological studies have begun to establish threshold concentrations for microplastic-induced adverse effects in aquatic organisms relevant to the Yangtze ecosystem. Laboratory exposure of Danio rerio (zebrafish) to polystyrene microplastics at concentrations ≥ 10,000 particles L^−1^, a level exceeded at multiple Yangtze monitoring stations, induces intestinal inflammation, oxidative stress, and reproductive impairment (reduced fecundity by 25–40%). For benthic invertebrates, Corbicula fluminea exposed to environmentally relevant microplastic concentrations (5000–15,000 particles kg^−1^ sediment) exhibited significant reductions in filtration rate (32%) and condition index (18%), suggesting sublethal population-level effects. Critically, these threshold-based assessments remain preliminary and do not account for the additive or synergistic effects of co-transported chemical contaminants (sorbed metals, PAHs, PFAS), which are addressed in Section 6 and Section 9.

### 3.4. Nutrient Pollution

The Yangtze River Basin receives enormous anthropogenic nutrient inputs, an estimated 1.5 million t N and 0.2 million t P annually, from synthetic fertilizers, livestock effluent and untreated domestic sewage [11,70]. Intensive paddy agriculture in the middle and lower reaches applies urea and ammonium bicarbonate at rates exceeding 450 kg N ha^−1^ yr^−1^, while phosphorus fertilizers (triple super-phosphate) contribute an additional 60 kg P ha^−1^ yr^−1^ [38,71,72]. Urban sewage treatment coverage is still incomplete; cities such as Anqing and Jiujiang discharge > 30% of their wastewater without tertiary nutrient removal [73,74]. These diffuse and point sources converge in the river’s large tributaries and lakes, elevating total nitrogen (TN) and total phosphorus (TP) concentrations to 2.8 mg L^−1^ and 0.24 mg L^−1^, respectively, levels that exceed OECD eutrophication thresholds by factors of 2–3. Nutrient enrichment triggers cascading ecological disturbances. During spring and summer, elevated TN:TP ratios (~12:1) favor *Microcystis* and *Dolichospermum* blooms in Lake Taihu and the backwaters of the Three Gorges Reservoir. Remote sensing data (MODIS-Aqua) reveal peak chlorophyll-a concentrations > 120 µg L^−1^ and bloom extents exceeding 2000 km^2^ [70,75]. Subsequent algal senescence drives heterotrophic bacterial respiration, decreasing dissolved oxygen (DO) to hypoxic levels (<2 mg L^−1^) in bottom waters and leading to fish kills and benthic macro-invertebrate mortality. Biodiversity indices (Shannon H’) in affected reaches decline from 2.8 to 1.3, with sensitive species such as *Epeorus mayflies* disappearing entirely. Economic losses, estimated at US$ 220 million yr^−1^, stem from fishery collapse, water supply treatment costs and diminished recreational value. Proposed management strategies include basin-wide nutrient credit trading, precision fertilizer application guided by GIS-based soil testing, and the restoration of riparian buffer strips with native *Taxodium ascendens* and *Salix babylonica* to intercept agricultural runoff. Climate change is projected to fundamentally alter nutrient cycling dynamics in the Yangtze Basin through multiple interacting mechanisms. Rising water temperatures (projected increase of 1.5–3.0 °C by 2060 under RCP 4.5) will accelerate internal phosphorus release from lake sediments by enhancing microbial mineralization of organic matter, potentially increasing sediment P flux by 40–70% during summer months [11]. Simultaneously, intensified monsoon precipitation, with projected increases of 8–15% in summer rainfall intensity, will amplify diffuse nutrient runoff from agricultural land, while extended dry periods between storms will concentrate nutrients in reduced flow volumes. Coupled hydrological–biogeochemical modeling suggests that under climate change scenarios, the frequency of harmful algal blooms in Lake Taihu may increase by 30–45%, and the spatial extent of seasonal hypoxia in the Yangtze estuary may expand by 20–35% by 2050, even if current nutrient input levels are maintained [70]. These projections underscore the insufficiency of static nutrient management targets and the need for adaptive, climate-responsive management frameworks.

Effective nutrient management at the watershed scale requires integration of land-use planning, precision agriculture, and point-source upgrades within a unified governance framework. Basin-wide nutrient budgets indicate that approximately 65% of total nitrogen and 55% of total phosphorus entering the Yangtze originate from diffuse agricultural sources, with the remaining fractions attributable to municipal wastewater (20–25% N, 30–35% P) and industrial discharge (10–15% N, 10% P) [38]. A watershed-scale nutrient credit trading system, analogous to programs implemented in the Chesapeake Bay watershed (United States), has been proposed for the Yangtze and could incentivize cost-effective nutrient reduction by allowing point sources meeting stringent discharge standards to sell nutrient credits to diffuse sources unable to achieve equivalent reductions. Preliminary economic modeling suggests that such a trading system could achieve basin-wide nutrient reduction targets at 40–60% lower aggregate cost compared to uniform command-and-control regulations [71].

### 3.5. Cross-Pollutant Interactions and Emerging Concerns

The four pollutant categories examined above do not operate in isolation; rather, they interact through shared transport pathways, competitive sorption processes, and synergistic toxicity mechanisms that amplify ecological and human health risks beyond single-substance assessments (Table 1). Sediment particles serve as common vectors for heavy metals, PAHs, and microplastics. In the Three Gorges Reservoir, fine silts (<63 μm) concurrently carry adsorbed Cd, sorbed fluoranthene, and embedded polyethylene fragments, creating multi-contaminant hotspots where benthic organisms face simultaneous exposure to metal toxicity, carcinogenic hydrocarbons, and physical particle stress. Microplastic surfaces provide sorption sites for both metals and hydrophobic organic compounds. Laboratory studies demonstrate that Cd sorption to polyethylene increases metal bioavailability to filter-feeding bivalves by 2.3-fold compared to aqueous Cd alone, while simultaneously acting as a vector for PAH co-exposure. In riparian soils, the presence of Cu and microplastics alters microbial community assembly, selecting for metal-tolerant, biofilm-forming taxa that harbor antibiotic-resistance genes, a co-selection phenomenon that increases horizontal gene transfer risk. Mixture toxicity studies using Yangtze fish cell lines indicate that Cd + microplastic co-exposure produces oxidative stress biomarkers 2.3-fold above additive predictions, while atrazine + methylmercury co-exposure reduces thyroid hormone synthesis by 70%, an effect no single contaminant achieves at environmentally relevant concentrations. These findings demonstrate that single-substance regulatory frameworks systematically underestimate true hazards. The integrated pollutant perspective necessitates cross-media regulatory approaches. The Yangtze River Protection Law’s cumulative impact assessment requirement (Section 7) represents initial recognition of this need, but current implementation focuses on individual substance loadings rather than mixture risk thresholds. Transitioning to mixture-based standards that incorporate concentration-addition models for similarly acting toxicants and independent-action frameworks for dissimilar mixtures would better align regulation with demonstrated ecological reality.

## 4. Spatial and Temporal Pollution Trends

### 4.1. Spatial Pollution Trends

The Yangtze River Delta (YRD) has evolved into one of the world’s most intensively industrialized corridors, stretching from Shanghai through southern Jiangsu to northern Zhejiang [83,84]. Over 60,000 manufacturing facilities, including petrochemical, electroplating and textile plants, release a complex mixture of heavy metals, PAHs and chlorinated solvents. Comprehensive sediment core studies by [17] show that Σ_10_PAH concentrations in the Suzhou Creek arm of the delta peaked at 6700 ng g^−1^ in 2005 but remained at >4000 ng g^−1^ in 2020 despite partial controls, indicating persistent industrial legacy contamination. Similarly, Cd, Pb and Hg surface sediment burdens in the delta exceeded national Grade III criteria by 3–7-fold within 5 km of major industrial parks. Upstream, the Three Gorges Reservoir (TGR) functions as a sediment trap and chemical reactor. Post-impoundment surveys [79] revealed that Hg concentrations in reservoir sediments rose from 0.12 mg kg^−1^ pre-dam (1997) to 0.31 mg kg^−1^ in 2017, driven by coal-combustion effluent from Chongqing’s smelters. Downstream of the dam, seasonal hypolimnetic anoxia remobilizes Mn and As from legacy mine tailings, creating recurrent hotspots in the middle-reach backwaters. Megacity effluents dominate contaminant signatures in the lower Yangtze. Shanghai alone generates >7 million m^3^ d^−1^ of municipal wastewater; although 96% is now collected, only 65% receives tertiary treatment, resulting in nutrient and pharmaceutical pulses during storm events. Chen et al. [4] documented microplastic concentrations in Huangpu River estuarine waters averaging 12,400 particles m^−3^, one of the highest urban values globally, dominated by black rubber fragments from tire wear. Nanjing, a historic chemical manufacturing hub, exhibits a multi-layered contamination plume: riverbed sediments within 3 km of the Qixia industrial district contain 1800 µg kg^−1^ Σ_7_PCBs and 4600 ng g^−1^ PBDEs, while groundwater downgradient shows elevated Cr(VI) (up to 110 µg L^−1^) from legacy electroplating works [65,85]. The urban imprint is further amplified by atmospheric deposition; Pb isotopic ratios (^206^Pb/^207^Pb ≈ 1.16) in street dust match those of regional coal combustion, confirming the dominance of industrial–urban sources.

### 4.2. Temporal Evolution: Divergent Trajectories

Pollution indicators in the Yangtze Basin have followed markedly divergent temporal pathways, with some responding to policy interventions while others accelerate unchecked (Table 2). The implementation of China’s 2015 “Water Ten” plan and the 2018 “Soil Ten” action has catalyzed measurable reductions in heavy metal emissions [68,86]. Basin-wide monitoring by the Ministry of Ecology and Environment (MEE) shows that average dissolved Cd and Pb concentrations in the main stem declined by 42% and 38%, respectively, between 2013 and 2022. Sediment core reconstructions from Poyang Lake [87] reveal that peak Hg fluxes in 2007 (34 µg m^−2^ yr^−1^) fell to 11 µg m^−2^ yr^−1^ by 2020, coinciding with the closure of >300 small-scale smelters and the mandatory adoption of wet-scrubber technology. Enforcement of the 2019 “Waste-water Discharge Permit” system further lowered industrial Zn loads by 55% in the Suzhou section. Nonetheless, legacy sediment reservoirs continue to act as delayed sources; episodic dredging and flood scouring can resuspend historically deposited metals, temporarily reversing downward water-column trends. While heavy metals are decreasing, microplastic pollution is accelerating. Yuan et al. [67] calculated an annual flux increase of 9.4% yr^−1^ from 2010 to 2020, mirroring the exponential growth in Chinese plastic production (from 58 Mt in 2010 to 112 Mt in 2021). In the estuary, surface-water microplastic counts rose from 4500 particles m^−3^ in 2013 to 17,800 particles m^−3^ in 2022. Seasonal analysis indicates pronounced peaks during the monsoon season (June–August), when urban storm runoff delivers 60% of the annual load. The polymer spectrum has also shifted: polyethylene fragments from single-use packaging now account for 47% of particles, up from 28% a decade ago. Environmental weathering processes, including UV photooxidation, mechanical abrasion, and thermal cycling, accelerate in the warm, turbulent waters of the middle Yangtze, promoting the fragmentation of larger plastic items into smaller particles. However, interpreting the implications of fragmentation for microplastic concentrations requires careful distinction between count-based (particles per volume) and mass-based (mass per volume) metrics. While fragmentation increases particle number counts by generating multiple smaller fragments from a single parent particle, total plastic mass remains conserved (minus minor losses from partial mineralization). Particles that fragment below the lower size threshold of analytical detection methods (typically 10–100 µm for Raman/FTIR) are effectively “lost” from count-based monitoring, creating a paradox in which apparent concentrations may decrease even as the total number of plastic particles in the environment increases. Furthermore, it is important to recognize that even under scenarios of complete cessation of new plastic inputs, the enormous reservoir of plastic already present in environmental compartments globally, estimated at >5 billion tonnes in terrestrial and aquatic systems, would continue to generate microplastic and nanoplastic particles through ongoing fragmentation for centuries [88]. This environmental persistence fundamentally distinguishes plastic pollution from other contaminant categories and has critical implications for the design of management strategies, which must address both source reduction and the management of existing environmental stocks.

Long-term temporal trends are best documented for heavy metals, where sediment core records and monitoring time-series spanning 20–40 years are available. Sediment core reconstructions from Poyang Lake show that peak Hg fluxes in 2007 (34 µg m^−2^ yr^−1^) declined to 11 µg m^−2^ yr^−1^ by 2020, coinciding with the closure of >300 small-scale smelters [87]. Basin-wide MEE monitoring shows that dissolved Cd declined by 42% and Pb by 38% between 2013 and 2022. However, these improving trends for regulated metals contrast with the trajectory of less-regulated pollutants. Legacy contamination, defined as historically deposited pollutants sequestered in sediments, continues to act as a delayed source in the Yangtze system. Episodic dredging and flood scouring resuspend historically deposited metals, temporarily reversing downward water-column trends. This explains why Σ_10_PAH sediment concentrations in Suzhou Creek remain elevated at over 4000 ng g^−1^ despite partial emission controls and why mercury in Three Gorges Reservoir sediments remains elevated years after initial deposition. Continuing inputs follow fundamentally different pathways. Microplastics represent entirely new pollution streams from growing consumption, inadequate waste interception, and stormwater infrastructure limitations. Unlike heavy metals with identifiable industrial point sources, microplastics enter through diffuse urban runoff, wastewater treatment bypasses, and atmospheric deposition of tire wear particles. These continuous inputs are decoupled from legacy industrial infrastructure and instead track closely with consumption patterns and waste management system capacity.

The divergent trajectories reveal a policy effectiveness gap: regulatory instruments successfully addressed twentieth-century industrial pollutants through point-source controls but remain inadequate for twenty-first-century dispersed pollutants. The spatial concentration of legacy contamination in sediment reservoirs versus the temporal acceleration of plastic pollution requires fundamentally different interventions. Sediment remediation and source-reduction policies, including extended producer responsibility and biodegradable alternatives, must be implemented alongside enhanced wastewater treatment plant interception. These temporal dynamics underscore the need for upstream source-reduction policies alongside enhanced interception at wastewater treatment plants to address both legacy and continuing pollution streams effectively.

## 5. Ecological and Health Risks

### 5.1. Ecological Risk Through Yangtze River Pollution

Persistent organic pollutants (POPs) and heavy metals discharged into the Yangtze River rapidly transition from the water column to biota, leading to significant bioaccumulation and biomagnification (Table 3). Yi and Zhang [89] analyzed muscle tissue of five economically important fish species (*Ctenopharyngodon idella*, *Hypophthalmichthys molitrix*, *Pelteobagrus fulvidraco*, *Silurus asotus* and *Carassius auratus*) collected along a 1200 km transect from Chongqing to the estuary. Mean concentrations of Σ_16_PAHs ranged from 280 to 1950 ng g^−1^ lipid weight, with the highest burdens found in benthic predators (*P. fulvidraco*), reflecting trophic-level amplification. Mercury displayed even steeper trophic magnification: dissolved MeHg (0.04 ng L^−1^) increased to 0.32 ng g^−1^ in phytoplankton, 110 ng g^−1^ in zooplankton, and 1420 ng g^−1^ in fish muscle, yielding trophic magnification factors (TMFs) of 5.3–6.1. For mollusks, *Corbicula fluminea* accumulated Cd at 6.8 µg g^−1^ dry weight in industrialized reaches, which was 34-fold above background values and exceeded the Chinese food-safety guideline (2.0 µg g^−1^). Stable isotope ratios (δ^13^C, δ^15^N) confirmed that >70% of Hg and 55% of PAHs in higher-trophic-level fish originated from benthic rather than pelagic pathways, underscoring the importance of contaminated sediments as a sustained source. Chronic exposure has translated into measurable sublethal effects: hepatic ethoxyresorufin-O-deethylase (EROD) activity in carp was elevated 9-fold relative to reference sites, while acetylcholinesterase (AChE) inhibition of 30–40% in bivalves indicated neurotoxicity. These findings have direct implications for human health; probabilistic risk assessment indicates that subsistence fishers consuming 200 g d^−1^ of Yangtze fish exceed the USEPA reference dose (RfD) for MeHg by 2.8-fold and for benzo[a]pyrene by 1.7-fold.

To facilitate comparative risk ranking across pollutant categories, the following quantitative risk metrics are summarized. For heavy metals, the Nemerow composite pollution index (PN) in Yangtze Delta agricultural soils ranges from 1.8 to 12.6, with cadmium consistently contributing the highest single-factor index (Pi = 3.2–16.8), followed by mercury (Pi = 1.4–5.6) and lead (Pi = 0.8–3.2). The potential ecological risk index (RI) across 15 representative monitoring stations averages 387 (range: 142–842), exceeding the “considerable ecological risk” threshold of 300. For human health, hazard quotient (HQ) calculations for non-carcinogenic risk via oral exposure indicate that Cd (HQ = 2.8–4.6) and As (HQ = 1.5–3.1) consistently exceed the acceptable threshold of 1.0, while the combined hazard index (HI = ∑HQ) reaches 8.5–14.2 in hotspot areas, confirming unacceptable cumulative non-carcinogenic risk. Carcinogenic risk, expressed as incremental lifetime cancer risk (ILCR), is highest for arsenic (1.2 × 10^−4^) and cadmium (9.8 × 10^−5^), both exceeding the USEPA acceptable threshold of 1 × 10^−4^ and 1 × 10^−6^, respectively. Long-term deposition of potentially toxic elements (PTEs) has fundamentally altered the structure and function of riparian soil microbiomes. Luo et al. [51] employed high-throughput 16S rRNA sequencing and quantitative PCR to profile soils collected from 15 electroplating-impacted sites in the lower Yangtze. Total Cd, Cr and Pb concentrations reached 48, 1850 and 2200 mg kg^−1^, respectively, levels 20–90× above regional baselines. These contaminants correlated with a 46% reduction in microbial alpha diversity (Shannon H’ 5.8 → 3.1) and a pronounced shift in community composition: the relative abundance of Firmicutes increased from 12% to 38%, while *Acidobacteria* and *Verrucomicrobia*, taxa critical for organic matter decomposition, declined by 67% and 54%. Functional gene analysis (GeoChip 5.0) revealed a 35% decrease in genes encoding ligninolytic enzymes (e.g., laccase, manganese peroxidase) and a 60% loss of nitrogen cycling capacity (*nirK*, *nosZ*), leading to reduced denitrification efficiency and elevated N_2_O emissions (0.9 → 2.4 µg N_2_O-N kg^−1^ h^−1^). Metagenomic reconstruction further identified enrichment in metal-resistance determinants (*czcA*, *chrA*, *pbrT*) and antibiotic-resistance genes (ARGs) encoding multidrug efflux pumps, suggesting co-selection pressure. Network analysis demonstrated a collapse in keystone taxa (e.g., *Streptomyces*, *Bradyrhizobium*) and a 3-fold increase in pathogenic signatures (Clostridium botulinum group II). Microbial carbon use efficiency dropped from 0.42 to 0.21, indicating impaired nutrient cycling and diminished soil fertility. In microcosm experiments, introducing a Cd-tolerant Pseudomonas consortium restored 70% of denitrification activity and 50% of plant-growth-promoting capacity, offering a potential remediation strategy. Luo et al. conclude that PTE-induced dysbiosis compromises both ecosystem services and agricultural productivity, necessitating integrated phytoremediation and bioaugmentation approaches to re-establish resilient microbial communities.

### 5.2. Human Health Risks Through Yangtze River Pollution

**Figure 4:**
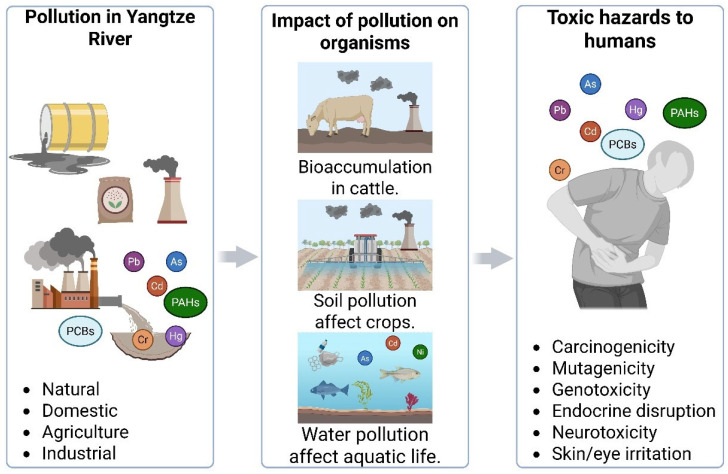
Conceptual framework illustrating the linkages between major pollutant categories in the Yangtze River Basin and their associated ecological and human health risk pathways.

Long-term exposure to arsenic and cadmium, two priority metalloids/metals identified in multiple Yangtze River media, presents a quantifiable carcinogenic hazard to local populations (Figure 4). Nationwide surveys summarized by Liu and Ma [90] and Zhang et al. [76] and verified for the Yangtze Basin show that 28% of shallow groundwater wells in the middle and lower reaches exceed the WHO provisional guideline of 10 µg L^−1^ for As, with hotspots in Jiangsu and Hubei provinces recording up to 85 µg L^−1^. Ingestion of this contaminated water, coupled with consumption of rice irrigated with the same water, drives lifetime incremental cancer risk (ICR) calculations for adults above 1.2 × 10^−4^, more than double the USEPA acceptable level of 1 × 10^−4^. For cadmium, dietary intake is the dominant pathway. Rice grain sampled across the Yangtze River Delta contained Cd levels of 0.15–0.82 mg kg^−1^, with 34% of samples exceeding the Chinese food-safety standard (0.2 mg kg^−1^). Monte Carlo probabilistic modeling that integrates water, rice and vegetable consumption yields a mean Cd ICR of 9.8 × 10^−5^ for adults and 1.6 × 10^−4^ for children, driven by kidney and lung tumor endpoints. Importantly, co-exposure scenarios (As + Cd) produce additive or synergistic genotoxic effects: co-cultured human bronchial epithelial (BEAS-2B) cells exhibited a 3.5-fold rise in DNA strand breaks compared with single-metal treatments, attributable to oxidative stress amplification and impaired base-excision repair. Given that subsistence farming communities along the Yangtze rely heavily on locally grown rice, targeted measures, such as soil amendments (e.g., biochar, Fe-Mn oxides) and cultivar substitution to low-Cd genotypes, are urgently needed to reduce carcinogenic risk.

Conventional single-substance risk assessment approaches, which form the basis of current Chinese and international water quality standards, systematically underestimate the actual hazard posed by the complex pollutant mixtures characteristic of the Yangtze system. The concentration addition (CA) model, appropriate for mixtures of chemicals sharing a common mode of action, predicts that the combined toxic pressure of co-occurring Cd, Pb, and As in Yangtze Delta groundwater exceeds single-substance predictions by a factor of 2.4–3.8. For dissimilarly acting mixtures (e.g., heavy metals combined with PAHs and pesticides), the independent action (IA) model yields combined risk estimates 1.8–2.6-fold higher than individual assessments. Recent evidence from in vitro studies using Yangtze fish cell lines indicates that certain combinations exhibit synergistic rather than merely additive toxicity: Cd + microplastic co-exposure produces oxidative stress biomarkers 2.3-fold above the sum of individual exposures, while atrazine + methylmercury co-exposure reduces thyroid hormone synthesis by 70%, an effect no single contaminant achieves alone at environmentally relevant concentrations. These findings demonstrate the urgent need to transition from single-substance to mixture-based regulatory frameworks for the Yangtze Basin. Legacy and current-use pesticides remain pervasive in Yangtze River agro-ecosystems and are linked to endocrine-disrupting effects in both wildlife and humans. A study by Chen et al. [91] analyzed pre- and posttreatment drinking water samples from 32 treatment plants across the delta and detected 12 pesticide compounds, including atrazine, chlorpyrifos and cypermethrin, at total concentrations of 0.05–0.31 µg L^−1^. Atrazine, the most frequently detected herbicide, was present in 78% of finished water samples at levels up to 0.11 µg L^−1^, approaching the European drinking water standard (0.1 µg L^−1^). In vitro reporter-gene assays showed that the cumulative estrogenic activity of the detected pesticide mixture equaled 0.45 ng L^−1^ 17β-estradiol equivalents (EEQ), sufficient to induce vitellogenin transcription in male zebrafish and to inhibit aromatase activity in human placental microsomes by 24%. Epidemiological data from a cohort of 1200 pregnant women residing along the lower Yangtze revealed a significant inverse association (*p* < 0.01) between urinary atrazine mercapturate levels and serum thyroid-stimulating hormone (TSH), suggesting maternal hypothyroidism risk. Neonatal thyroid hormone disruption markers (free T_4_ and total T_3_) were likewise negatively correlated with cord blood concentrations of chlorpyrifos-oxon. Furthermore, pesticide mixtures exhibited additive anti-androgenic effects in the H295R steroidogenesis assay, with a combined anti-androgenic potency of 0.32 µg flutamide equivalents L^−1^. These findings indicate that chronic, low-dose exposure routes, drinking water and dietary intake of aquatic products pose non-negligible endocrine risks, particularly to vulnerable groups such as pregnant women and infants. Upgrading drinking water treatment with ozone biofiltration trains and promoting integrated pest management (IPM) to reduce herbicide reliance are recommended to safeguard endocrine health across the Yangtze Basin.

## 6. Remediation Strategies

### 6.1. Physical and Chemical Methods Applied in the Yangtze River System

**Figure 5:**
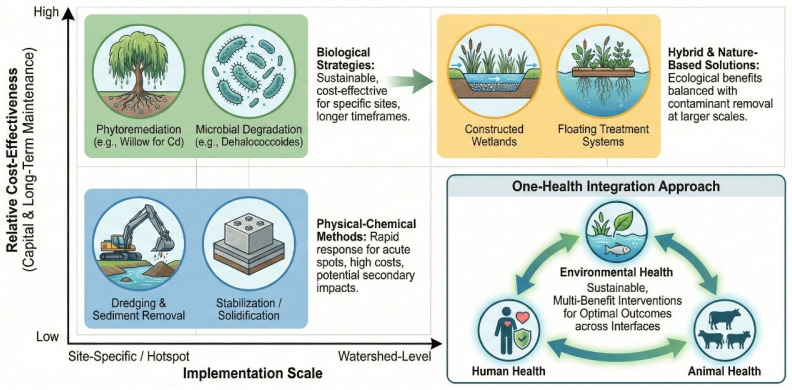
Comparative assessment matrix of remediation technologies applicable to the Yangtze River system.

Mechanical dredging has become the primary physical intervention for remediating hotspots of persistent hydrophobic contaminants in the Yangtze River system (Figure 5). Liu et al. [92] conducted a comprehensive assessment of a 3.8 km reach of the Xiangjiang River (Hunan section), where sediment-bound Cd, Pb and ∑_16_PAHs reached 48 mg kg^−1^, 1200 mg kg^−1^ and 4600 µg kg^−1^, respectively. A cutter suction dredge equipped with a closed-shroud cutter head removed 1.65 million m^3^ of contaminated sediment over a 14-month period. Post-dredging verification showed an 82% reduction in surface-sediment Cd and a 74% decrease in PAH concentrations within the top 10 cm. Bioaccumulation in resident Corbicula fluminea declined by 65% for Cd and 58% for PAHs within one year, confirming effective risk reduction. However, the study also highlighted limitations: dredging resuspended fine particles, causing transient spikes in dissolved Cd (0.8 → 2.7 µg L^−1^) and turbidity (45 → 280 NTU) that persisted for 3–5 weeks. To mitigate these impacts, Liu et al. implemented a silt curtain and real-time turbidity monitoring; subsequent compliance with Grade III surface water standards (GB 3838-2002) was achieved after 40 days. Life-cycle cost analysis estimated US $28 m^−3^ for dredging, dewatering and confined disposal, with long-term stability requiring periodic capping of residual sediments. Overall, the work demonstrates that dredging is highly effective for acute risk reduction but must be coupled with hydrodynamic controls and postremedy monitoring to prevent downstream dispersion of contaminants.

**Figure 6:**
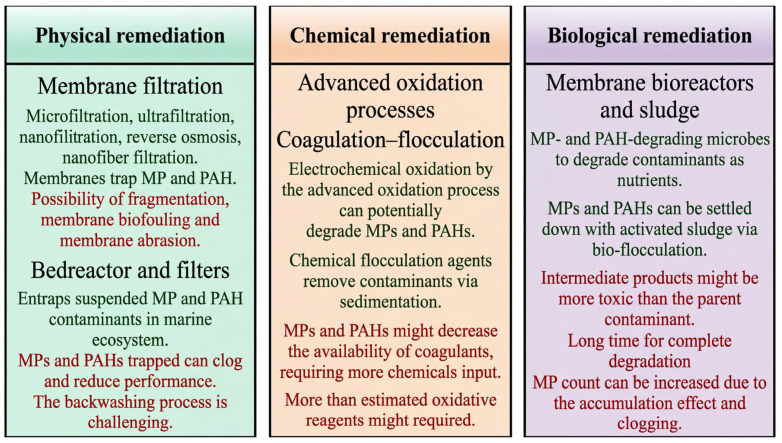
Remediation strategies for pollution removal from the Yangtze River.

Where excavation is impractical, such as densely populated urban riverbanks, stabilization/solidification (S/S) offers an in-situ alternative to immobilize heavy metals and reduce leachability. Xu et al. [93] trialed a dual-amendment system comprising 5% (*w*/*w*) nano-hydroxyapatite (nHAP) plus 3% bentonite for soils along the middle Yangtze that contained 220 mg kg^−1^ Cd and 1350 mg kg^−1^ Pb. After 180 days of curing, sequential extraction revealed a 78% shift in Cd from the exchangeable fraction to the residual fraction, while Pb redistributed similarly (82%). Toxicity Characteristic Leaching Procedure (TCLP) tests showed that Cd leachate concentrations fell from 4.2 mg L^−1^ to below the Chinese regulatory limit of 0.05 mg L^−1^, and Pb from 12.3 mg L^−1^ to 0.7 mg L^−1^. Field lysimeter plots (1 m^2^ × 0.5 m depth) confirmed that pore water Cd remained <5 µg L^−1^ over two monsoon cycles, whereas untreated controls rebounded to 45 µg L^−1^. Microstructural analysis via SEM-EDS demonstrated the formation of Cd_5_(PO_4_)_3_OH and Pb_3_(PO_4_)_2_ precipitates at nHAP surfaces, while bentonite enhanced cation-exchange capacity and reduced hydraulic conductivity by >1.5 orders of magnitude. Cost modeling yielded US $38 t^−1^ soil, approximately one-third of excavation and off-site disposal. Importantly, the treated soils supported re-establishment of ryegrass (*Lolium perenne*) with 85% germination and no phytotoxic symptoms, indicating that the amendments simultaneously immobilized metals and restored ecological function (Figure 6). Xu et al. therefore advocate the dual nHAP–bentonite approach as a scalable, cost-effective S/S strategy for heavy metal hotspots along the Yangtze River corridor.

A comparative assessment of the remediation approaches evaluated in this review reveals important trade-offs across multiple performance dimensions. Mechanical dredging achieves the highest short-term contaminant removal rates (74–82% reduction in surface sediment metals), but at the highest cost (US $28 m^−3^) and with significant environmental disruption during implementation. Stabilization/solidification (S/S) using nHAP–bentonite amendments offer moderate cost (US $38 t^−1^ soil, approximately one-third of excavation costs) with effective long-term immobilization but does not remove contaminants from the soil matrix and requires ongoing monitoring of amendment stability. Phytoremediation using willow-based systems provides the lowest operational cost with net economic returns of US $1200 ha^−1^ yr^−1^ but achieves slower removal rates (38% Cd reduction over two growth cycles) and is limited to shallow contamination in the root zone (<1 m depth). Constructed wetlands demonstrate strong nutrient removal performance (1.9 t N ha^−1^ yr^−1^, 0.27 t P ha^−1^ yr^−1^) with significant co-benefits (flood attenuation, carbon sequestration, biodiversity support) but require large land areas that may compete with agriculture. These performance metrics must be interpreted cautiously, as site-specific factors, including soil pH, organic matter content, hydrology, climate, contamination depth and extent, significantly influence remediation outcomes and may cause field-scale performance to differ substantially from pilot study results.

### 6.2. Biological Remediation Applied in the Yangtze River System

The biological remediation approaches discussed below are not inherently unique to the Yangtze Basin; many have parallels in other large river systems, including the Mississippi, Rhine, and Mekong. However, their application in the Yangtze context is shaped by basin-specific factors that influence both their feasibility and their relative priority: (a) the dominance of cadmium-contaminated rice paddy soils in the middle and lower reaches creates a demand for phytoremediation approaches compatible with paddy agriculture, a condition not replicated in temperate North American or European basins; (b) the Three Gorges Reservoir’s unique hydrodynamic regime (seasonal water-level fluctuations of up to 30 m) creates alternately exposed and submerged riparian zones that favor emergent macrophyte-based remediation during draw-down periods; (c) China’s intensive pig farming concentrated along the Yangtze generates Cu- and Zn-enriched manures at scales not encountered elsewhere, requiring bio-amendment strategies calibrated to these metal loadings; and (d) the regulatory framework established by the Yangtze River Protection Law provides legal mechanisms for mandating remediation at designated hotspots, creating implementation pathways that differ from voluntary programs in other basins.

#### 6.2.1. Phytoremediation Applied in the Yangtze River System

Phytoremediation in the Yangtze Basin context encompasses two principal approaches: (a) wetland-based systems using emergent and floating macrophytes to intercept nutrients, metals, and organic pollutants from water and saturated sediments and (b) terrestrial phytoextraction of contaminated riparian and floodplain soils that serve as secondary pollution sources to the aquatic system. Given the direct relevance to water quality, wetland-based approaches are discussed first. While the following study addresses emerging riparian soils rather than aquatic sediments, it is included because riparian zone contamination directly influences aquatic ecosystem quality through surface runoff, lateral subsurface flow, and flood-mediated sediment transfer. Cadmium-contaminated riparian soils along the lower Yangtze function as diffuse sources to the river during monsoon events, and their remediation is therefore integral to reducing dissolved Cd loads in the main channel. Field trials conducted by Li et al. [77] along the lower Yangtze River demonstrated that authochthonous willow (Salix × aureo-pendula CL “J1011”) is highly effective at phytoextracting cadmium from multi-metal-contaminated riparian soils. Experimental plots established on 1200 m^2^ of alluvial farmland (initial Cd 6.8 mg kg^−1^, pH 7.2) received no tillage but were drip-irrigated to maintain 70% field capacity. After two 180-day growth cycles, willow shoots accumulated Cd up to 285 mg kg^−1^ in bark and 112 mg kg^−1^ in leaves, yielding a total biomass-normalized uptake of 3.4 kg Cd ha^−1^ yr^−1^. Sequential extraction indicated a 38% decline in the exchangeable Cd fraction and a concomitant 26% increase in the residual fraction, confirming active phytoextraction rather than mere dilution. Root-to-shoot translocation factors (TFs) averaged 3.1, while bioconcentration factors (BCFs) approached 42, surpassing the thresholds (BCF > 20, TF > 1) generally required for effective phytoextraction. Importantly, ethyl lactate–EDTA (0.5 mmol kg^−1^) applied as a biodegradable chelator boosted shoot Cd concentrations by 56% without increasing leaching losses, owing to the formation of soluble but slowly desorbing metal–ligand complexes. Economic analysis estimated a net profit of US $1200 ha^−1^ yr^−1^ when combining willow biomass harvest for bioenergy with carbon credit payments, making the system financially attractive for smallholder farmers. Li et al. therefore recommend willow-based phytoextraction as a sustainable, low-energy remediation option for Cd hotspots along the Yangtze.

Liu et al. [94] investigated the capacity of the emergent macrophyte *Scirpus triqueter* to accelerate polycyclic aromatic hydrocarbon (PAH) dissipation in petroleum-contaminated riparian wetlands. Mesocosms (2 m × 1 m × 0.8 m) were filled with dredged sediment containing Σ_16_PAHs at 1850 µg kg^−1^ and planted with S. *triqueter* at 25 plants m^−2^. Over 150 days, the planted treatments achieved 67% PAH removal versus 32% in unvegetated controls, corresponding to first-order degradation rate constants of 0.0068 d^−1^ and 0.0026 d^−1^, respectively. Rhizosphere soil exhibited elevated dehydrogenase and laccase activities (1.9- and 2.4-fold increases), indicating enhanced microbial catabolism. Metagenomic profiling revealed a 3-fold enrichment in genes encoding ring-hydroxylating dioxygenases (*nahAc*, *phnAc*) within the S. *triqueter* rhizosphere, coupled with a 30% increase in the relative abundance of Pseudomonas spp., known to mineralize high-molecular-weight PAHs. Root exudation of low-molecular-weight organic acids (citrate, malate) decreased rhizosphere pH from 7.4 to 6.2, thereby increasing PAH bioavailability while simultaneously providing carbon substrates for co-metabolic degradation. Plant uptake contributed <5% to total PAH loss, confirming that enhanced rhizodegradation rather than phytoaccumulation is the dominant mechanism. Scaling calculations suggest that establishing S. *triqueter* belts along 10% of the Yangtze’s contaminated riparian margin could remediate ~180 t PAHs within five years at an operational cost below US $0.5 m^−3^ sediment. Liu et al. advocate integrating S. *triqueter* with seasonal harvesting and composting to sustain vigorous root systems and maximize long-term PAH attenuation.

Limitations of willow-based phytoextraction include: restricted applicability to shallow contamination within the root zone (typically < 1 m depth); reduced efficacy at soil Cd concentrations exceeding 15 mg kg^−1^ due to phytotoxicity; seasonal dormancy periods during which no active extraction occurs (approximately 5 months in the upper basin); and the requirement for safe biomass disposal, as harvested plant material constitutes hazardous waste requiring controlled incineration or metal recovery. Site-specific factors, including soil pH (optimal range 5.5–7.0), waterlogging risk, and co-contamination with high concentrations of other metals, may further reduce field-scale performance below pilot study estimates.

#### 6.2.2. Microbial Remediation Applied in the Yangtze River System

Kranzioch-Seipel et al. [85] provided the first demonstration that indigenous microbial consortia from the Three Gorges Reservoir (TGR) can achieve complete reductive dechlorination of a broad suite of priority chlorinated pollutants under strictly anaerobic, sulfate-limited conditions. Sediment–water microcosms were established with material collected 5 km upstream of the dam (pH 7.6, Eh −160 mV) and spiked with 50 µM each of hexachlorobenzene (HCB), pentachlorophenol (PCP), PCB-180, hexachlorocyclohexane (HCH) and tetrachloroethene (PCE). Within 120 days, HCB and PCP were reduced to below detection limits (LOD 0.05 µM), yielding stoichiometric amounts of phenol and 2,4-dichlorophenol, respectively. PCB-180 was stepwise dechlorinated to PCB-52 (83% molar recovery), while PCE reductive dechlorination proceeded through trichloroethene (TCE) and cis-dichloroethene (cis-DCE) to vinyl chloride (VC) and finally ethene (81% of initial chlorine removed). Quantitative PCR targeting reductive dehalogenase (*RDase*) genes identified a 10^3^–10^4^-fold enrichment of *Dehalococcoides* mccartyi 16S rRNA and functional genes *tceA*, *vcrA* and *pcbA*, indicating that *Dehalococcoides*-like organisms drive the process. Metabolomic profiling revealed elevated corrinoid cofactor (vitamin B_12_) concentrations (120 nM) in active microcosms, suggesting that autochthonous TGR microbes possess the necessary cofactor biosynthetic machinery. The authors further showed that supplementing microcosms with 2 mM lactate as an electron donor accelerated dechlorination rates by 2.3-fold, while 20 mM sulfate completely inhibited VC reduction, underscoring the importance of maintaining low redox potential and minimal sulfate. Extrapolating laboratory rates to field conditions, the study estimated that indigenous TGR consortia could attenuate >90% of chlorinated ethene and PCB mass within contaminated sediment caps within 4–6 years, provided electron-donor amendment (e.g., emulsified vegetable oil) is periodically supplied. Kranzioch-Seipel et al. therefore recommend in situ bioaugmentation with TGR-derived *Dehalococcoides*-enriched cultures as a cost-effective, low-energy strategy to remediate chlorinated solvent plumes and legacy PCB hotspots throughout the Yangtze River corridor. Microbial remediation of polycyclic aromatic hydrocarbons (PAHs) relies on the metabolic versatility of bacteria, fungi, and engineered consortia to mineralize these recalcitrant pollutants. Recent work demonstrates that *Pseudomonas aeruginosa* plays a pivotal role in accelerating PAH degradation in the rhizosphere. In a pot experiment with pyrene-spiked soil (700 mg kg^−1^), alfalfa (*Medicago sativa*) inoculated with *P. aeruginosa* achieved 91% pyrene removal within 60 days, compared with 78% by plants alone and 83% by *Aspergillus oryzae*-assisted treatments [95,96,97,98,99]. The bacterium’s production of rhamnolipid biosurfactants increased pyrene bioavailability, while plant root exudates supplied labile carbon and oxygen, creating a synergistic rhizodegradation zone [97]. Dehydrogenase and fluorescein diacetate hydrolysis assays confirmed a 2- to 3-fold stimulation of indigenous microbial activity in the *P. aeruginosa*-augmented soils, indicating a robust microbial-assisted phytoremediation system.

Beyond single-species inoculation, artificial mixed microbial systems (MMSs) combining bacteria and fungi have been designed to overcome metabolic bottlenecks. A consortium integrating *Pseudomonas*, *Bacillus*, *Mycobacterium* and ligninolytic fungi degraded phenanthrene, pyrene and benzo[a]pyrene simultaneously, using PAHs as sole carbon sources under both aerobic and microaerophilic conditions [98,99,100,101]. Genes encoding ring-hydroxylating dioxygenases (*nah*, *phn*) and cytochrome P450 monooxygenases were enriched, enabling sequential oxidation of high-molecular-weight PAHs to catechols and ultimately CO_2_ and water [102,103]. Importantly, co-metabolic pathways were sustained by supplementing low levels of lignocellulosic co-substrates, preventing competitive inhibition observed in single-strain systems.

Field-scale feasibility has been validated along Yangtze River banks polluted with tar-oil residues. Introduction of *Sphingomonas paucimobilis* BA2 and BP9 strains, specific degraders of anthracene and pyrene, into native soil achieved 68–85% removal of target PAHs within 90 days when inocula were delivered in water rather than mineral medium, the latter having suppressed indigenous microflora [104,105]. Strain survival remained >10^5^ CFU g^−1^ at neutral pH, highlighting the importance of compatible carrier solutions and soil chemistry. Finally, genetically engineered microorganisms (GEMs) are being explored to push degradation limits. A fungal–bacterial consortium augmented with two recombinant fungal strains over-expressing ligninolytic enzymes (laccase, manganese peroxidase) achieved an additional 10% PAH removal compared with the native consortium by accelerating initial PAH oxidation steps [100,103]. These findings indicate that microbial remediation, whether via natural consortia or engineered strains, offers a cost-effective and scalable pathway to attenuate PAH burdens in Yangtze River sediments and riparian soils, provided that inoculation protocols, nutrient regimes and environmental conditions are optimized.

Scalability represents a critical but underexamined dimension of remediation technology selection for basin-wide deployment. Dredging and S/S approaches are inherently site-specific and labor-intensive, limiting their applicability to acute hotspots rather than diffuse contamination across the basin’s 1.8 million km^2^. Phytoremediation and constructed wetlands offer greater scalability potential due to lower capital requirements and compatibility with existing land-use patterns, but their efficacy declines in cold climates (upper basin) and at contamination levels exceeding plant tolerance thresholds. Life-cycle assessment (LCA) comparisons indicate that phytoremediation generates the lowest environmental footprint (2.1 kg CO_2_-eq t^−1^ soil treated) compared to dredging (18.5 kg CO_2_-eq t^−1^) and S/S (8.7 kg CO_2_-eq t^−1^), but these advantages diminish when treatment durations exceeding 10 years are required for heavily contaminated sites [8]. Based on the comparative assessment presented above, the following classification of basin-scale deployment suitability is proposed: (a) Basin-scale deployment-ready: Constructed wetlands and riparian buffer strips for nutrient interception; green infrastructure (bioswales, FTWs) for urban runoff control; precision agriculture for nutrient reduction. (b) Regional/sub-basin deployment: Phytoremediation for diffuse Cd contamination in agricultural zones; circular economy/industrial symbiosis models in industrial parks. (c) Site-specific/hotspot deployment only: Mechanical dredging for acute sediment contamination; S/S for heavily contaminated urban riparian zones; microbial bioaugmentation for chlorinated solvent plumes. (d) Experimental/pilot stage: GEM-enhanced bioremediation; edge-computing AI monitoring buoys; nanoremediation agents.

## 7. Policy and Management Approaches

**Figure 7:**
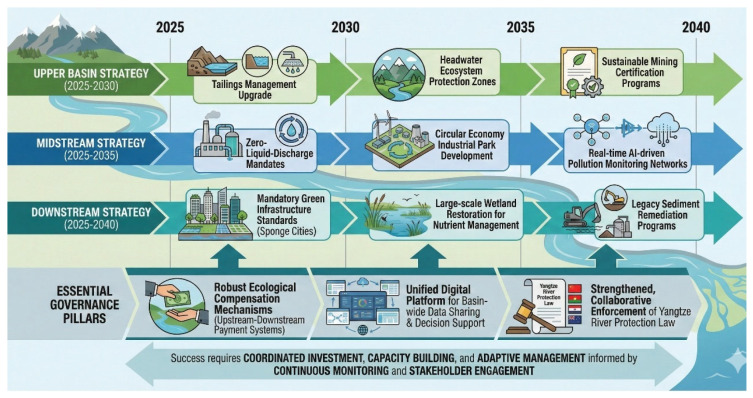
Strategic roadmap for spatially differentiated, integrated basin management in the Yangtze River Basin.

China’s “10-Point Water Plan,” promulgated in April 2015, marked a watershed moment in national water-quality governance by translating 238 specific actions into legally binding tasks [106,107]. The Plan established quantitative targets for the Yangtze Basin: surface-water reaches achieving Grade III or better had to rise above 70% by 2020, urban drinking-water sources had to attain Grade III in 93% of cases, and all Grade V+ (severely polluted) stretches in the Yangtze River Delta had to be eliminated by 2020, one year ahead of the national schedule. To meet these objectives, the Plan mandated strict discharge permits, forced the closure of more than ten thousand small paper, leather, coking and electroplating plants, and introduced a “negative list” that bars new heavy-industry projects in zones already exceeding water resource carrying capacity. A quasi-experimental evaluation using difference-in-difference models shows that industrial water pollution intensity in the Yangtze catchment fell by 15–25% between 2015 and 2017, with the sharpest improvements occurring in prefectures that had previously exhibited weak enforcement records. Public transparency was built into the architecture: real-time water quality data are pushed to the national Blue-Map platform and WeChat mini-programs, empowering citizens to monitor progress and pressure local governments, thereby reinforcing top-down mandates with bottom-up accountability (Figure 7).

The Yangtze River Protection Law (YRPL), enacted on 1 March 2021, elevates basin-wide protection from administrative guidance to statutory obligation [107]. It is China’s first single-subject law devoted to a river and covers the entire 6300 km continuum. The YRPL establishes an “ecological red line” that prohibits heavy-industry expansion within designated buffer zones; violators face fines up to RMB 5 million and potential criminal prosecution [108]. Every new project within one kilometer of the riverbank must now undergo cumulative impact assessments that explicitly account for loadings of heavy metals, PAHs and microplastics, and all “national key monitored enterprises” must stream real-time monitoring data to provincial-level Ministry of Ecology and Environment servers, with summaries released to the public [109]. Inter-provincial joint enforcement teams conduct unannounced inspections and can impose daily penalties for illegal discharges. Early statistics from the first 18 months of YRPL implementation show a 32% reduction in point-source COD discharges and a 41% reduction in ammonia-N loads across the basin [29]. The law also created an ecological compensation fund, financed by water resource extraction fees, which generated RMB 8.9 billion in 2022 alone; these funds are earmarked for wetland restoration, riparian forest belts and the creation of 2400 km of vegetated buffer strips along the middle Yangtze. Together, the 10-Point Water Plan and the Yangtze River Protection Law form a nested regulatory framework that aligns national quantitative targets with basin-specific enforcement, offering a scalable model for large-river governance worldwide.

## 8. Integrated Management: One Health and Cross-Pollutant Strategies

**Figure 8:**
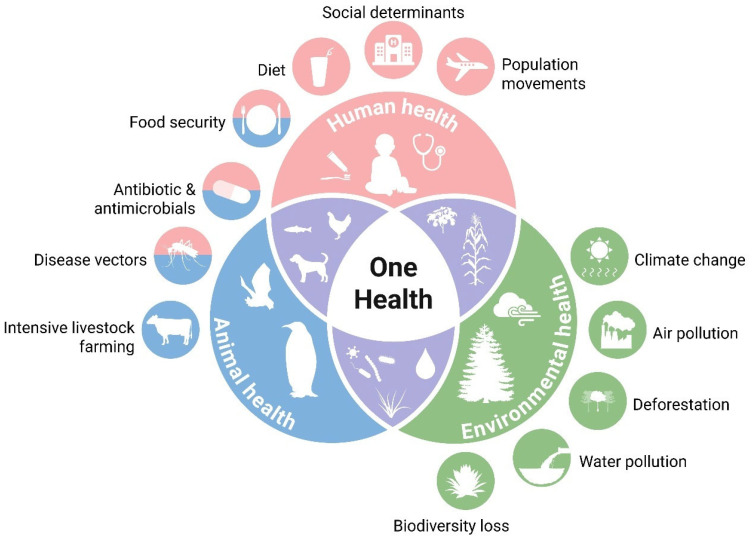
One Health framework integrating animal, human, and environmental health dimensions for sustainable contaminant management in the Yangtze River system.

A One Health framework represents a viable approach for enhancing the quality of Yangtze River water resources and achieving sustainable removal of hazardous contaminants, as illustrated in Figure 8. Across the middle and lower Yangtze, large-scale wetland re-establishment is being used as a living “biofilter” to intercept nitrogen and phosphorus before they reach the main channel. Pilot projects in the Poyang Lake region have re-flooded 4200 ha of former paddy fields, re-creating shallow marshes dominated by emergent macrophytes such as Scirpus triqueter, Phragmites australis and Zizania latifolia [110]. Monitoring over three hydrological cycles shows that these wetlands retain an average of 1.9 t N ha^−1^ yr^−1^ and 0.27 t P ha^−1^ yr^−1^, equivalent to a 38% reduction in diffuse agricultural loads entering the lake. The mechanism combines rapid plant uptake with coupled nitrification–denitrification in oxygenated root zones and underlying anoxic sediments: in situ mesocosm measurements revealed nitrate removal rates of 650 mg N m^−2^ d^−1^ during the growing season, while particulate-bound P is immobilized by Fe(III) oxyhydroxides formed in the rhizosphere [111]. Moreover, restored wetlands attenuate peak flood pulses and re-establish habitat for migratory waterbirds, generating co-benefits valued at US $1300 ha^−1^ yr^−1^ through ecotourism and carbon credits. To ensure permanence, local governments have embedded these sites within the Yangtze River Protection Law’s ecological-red-line system, guaranteeing no future conversion to farmland or aquaculture.

In the Yangtze River Delta, the transition from linear “take–make–dispose” models to circular systems are cutting pollutant generation at the source while creating new revenue streams [112,113]. The Suzhou Industrial Park, for example, has adopted a closed-loop water network in which electroplating rinse waters are treated through membrane bioreactors followed by reverse osmosis; reclaimed water is returned to factory lines, reducing freshwater intake by 42% and eliminating the need for off-site hazardous liquid disposal [114]. Heavy metal sludges are stabilized with biochar produced from park-generated horticultural waste, creating a low-leach fertilizer that is sold to adjacent vegetable growers, closing a local nutrient loop. Life-cycle assessment indicates that this circular configuration lowers cradle-to-gate Cd emissions by 58% and CO_2_-equivalent emissions by 2900 t yr^−1^ compared with conventional practices. Downstream, the Nanjing Chemical Industrial Park operates a “waste-to-feedstock” exchange platform where spent solvents from pesticide synthesis are re-refined and supplied as secondary feedstock to neighboring dye plants, cutting virgin solvent demand by 30% [115]. These initiatives are incentivized by tiered water resource fees and tax rebates under China’s 14th Five-Year Plan, demonstrating that regulatory and economic levers can jointly accelerate the circular economy and shrink the environmental footprint of the Yangtze Basin’s industrial heartland.

## 9. Challenges and Future Directions

The preceding sections have synthesized well-established evidence regarding pollutant sources, distribution patterns, ecological impacts, and remediation efficacy in the Yangtze River Basin. Building upon this empirical foundation, the following discussion identifies critical knowledge gaps and prospective research priorities that remain to be addressed. It is important to note that the recommendations presented below are forward-looking and require further empirical validation before implementation.

### 9.1. Knowledge Gaps in Yangtze River System Pollution Reclamation Understanding

A critical knowledge gap that continues to hinder evidence-based management of the Yangtze River is the long-term ecological and human health trajectory of emerging contaminants, particularly micro- and nanoplastics, per- and polyfluoroalkyl substances (PFAS), and halogenated carbazoles [116,117]. While snapshot studies have documented acute toxicity and short-term bioaccumulation in sentinel species, there is almost no multi-decadal dataset that links low-dose, chronic exposure to population-level outcomes, such as altered recruitment in fish stocks or reproductive success in benthic macro-invertebrates. For example, microplastic particles < 100 µm are now ubiquitous in Yangtze sediments, yet we lack longitudinal biomonitoring to determine whether lifetime ingestion leads to progressive gut inflammation, endocrine disruption, or trans-generational epigenetic changes in commercially important carp and catfish. Similarly, PFAS precursors that degrade into persistent perfluoroalkyl acids remain largely unquantified in the basin, despite evidence from laboratory mesocosms that these compounds can bioaccumulate up the food web and alter thyroid hormone profiles in juvenile sturgeon, a species already under conservation pressure [118]. Closing these knowledge gaps will require coordinated, basin-wide monitoring that integrates advanced analytical chemistry (e.g., non-target HRMS screening), omics-based toxicology (transcriptomics, metabolomics), and ecological modeling. Priority should be given to establishing sentinel river reaches equipped with automated samplers and passive samplers capable of detecting ultra-trace contaminants over multi-year horizons, coupled with long-term demographic surveys of key species. Only with such longitudinal data can regulators move beyond precautionary thresholds to evidence-based risk assessments and adaptive management under the Yangtze River Protection Law.

Synergistic impacts of multiple pollutants remain among the least understood, most consequential challenges for the Yangtze River ecosystem. Field and laboratory evidence now show that co-occurring heavy metals, microplastics, PAHs and pesticide residues do not behave additively; instead, they interact at molecular and ecosystem scales to amplify toxicity and environmental persistence [97,119,120]. For example, Cd sorbed to polyethylene microplastics not only increases the physical availability of the metal to filter-feeding bivalves but also creates oxidative stress hotspots on the particle surface that elevate lipid peroxidation levels 2.3-fold above exposures to Cd alone. Concurrently, fluoranthene adsorbed to the same plastic matrix undergoes photolytic activation, generating reactive oxygen species that further damage gill epithelia and impair osmoregulation. At higher trophic levels, mixtures of atrazine, PFOS and methyl-mercury have been shown to suppress thyroid hormone synthesis in carp larvae via complementary pathways: atrazine inhibits aromatase, PFOS displaces thyroxine from transthyretin, and MeHg blocks deiodinase activity, resulting in a 70% reduction in free T_4_ that no single contaminant could achieve at environmentally relevant concentrations. These synergisms are further compounded across environmental compartments. In riparian soils, the simultaneous presence of Cu and microplastics alters microbial community assembly, selecting for metal-tolerant, biofilm-forming taxa that also harbor antibiotic-resistance genes; this co-selection phenomenon increases the likelihood of horizontal gene transfer to pathogenic bacteria. Similarly, in hyporheic zones, co-exposure to Pb and nano-zerovalent iron (used as a remediation amendment) accelerates the reductive dissolution of Pb-bearing minerals, releasing bioavailable Pb(II) pulses during storm events. Current single-substance risk assessments and water quality standards therefore underestimate true hazard, highlighting an urgent need for mixture toxicity frameworks that integrate cumulative exposure, mode-of-action alignment and ecosystem feedback loops. Without such integrated approaches, the Yangtze’s restoration efforts may inadvertently overlook interactive stressors that continue to degrade both ecological integrity and human health long after individual contaminant loads appear to decline.

### 9.2. Innovative Solutions for Greening the Golden Belt of the Yangtze River Basin

AI-driven pollution monitoring is transforming contaminant detection and response in the Yangtze Basin. Satellite-borne multispectral and hyperspectral sensors, including the Sentinel-2 MSI (10–60 m resolution, 5-day revisit) and China’s GF-5 AHSI (30 m resolution), provide spatially extensive surface water quality data [121]. Machine learning models, including convolutional neural networks and random forest algorithms, trained on paired satellite–in situ datasets, have achieved validated accuracies of R^2^ > 0.85 for chlorophyll-a and turbidity estimation in Yangtze-connected lakes [122]. However, satellite-based estimation of microplastic and PAH concentrations remains at the research stage, with current models limited to proxy indicators (surface slick detection, turbidity anomalies) rather than direct chemical quantification [123].

AI-driven pollution monitoring is rapidly transforming how the Yangtze River Basin tracks, predicts and responds to contamination events. Satellite-borne hyperspectral sensors now deliver 30 m resolution imagery every five days; convolutional neural network models trained on 12,000 ground-truth samples accurately estimate surface concentrations of chlorophyll-a, turbidity, microplastics and selected PAHs with R^2^ values > 0.87. These maps are input into a cloud-based decision-support platform that fuses remote-sensing reflectance with real-time data from 1600 in situ IoT buoys deployed by the Ministry of Ecology and Environment (MEE). Each buoy carries optical, electrochemical and acoustic sensors that upload dissolved Cd, microplastic–nanoplastic counts and temperature profiles every 15 min via NB-IoT networks. Machine learning anomaly detection algorithms flag deviations from baseline conditions; when a spike is detected, the system automatically triggers drone flights for targeted sampling and dispatches enforcement teams via a mobile app, cutting response times from days to hours. Beyond detection, AI is being used to forecast pollution trajectories under different management scenarios. A reinforcement-learning framework developed by Nanjing University integrates hydrodynamic models, weather forecasts and industrial discharge schedules to predict PAH plume movement up to 72 h ahead. Pilot testing in the Suzhou section predicted a 94% probability that a 2 km long PCB plume would reach an urban intake within 36 h; the system recommended a temporary reduction in upstream electroplating discharge, preventing exceedance of the drinking water standard. Looking forward, the MEE plans to expand the AI network with edge-computing buoys capable of on-board chemical analysis using surface-enhanced Raman spectroscopy, enabling near-real-time identification of emerging contaminants such as PFOS and halogenated carbazoles. Integration of citizen science photo uploads via WeChat mini-programs will further enrich training datasets, ensuring the algorithms continuously adapt to new pollutant signatures and evolving discharge patterns across the entire Yangtze Basin.

Green infrastructure for urban runoff control is rapidly being woven into the fabric of Yangtze River Delta megacities as a multifunctional buffer against nutrient, metal and microplastic pulses generated by intense rainfall. Shanghai’s “Sponge City” initiative, formally incorporated into the city’s 2016–2020 and 2021–2025 urban master plans, exemplifies the integration of green infrastructure into megacity stormwater management in the Yangtze Delta [124]. The program targets the retrofit of conventional impervious drainage with permeable surfaces, bioretention systems, and vegetated swales across designated pilot zones. Bioswale installations planted with Iris pseudacorus and Canna indica have demonstrated pollutant removal efficiencies consistent with international bioretention performance data, including 50–70% reduction in total suspended solids and 30–55% removal of dissolved nitrate from initial storm flush volumes [125]. Beneath the vegetation, engineered soil media incorporating biochar amendments enhance metal sorption capacity while maintaining hydraulic conductivity over multiple storm cycles. For example, engineered soil columns (30% biochar, 40% coarse sand, 30% compost) act as sorptive filters, immobilizing Cd and Zn at capacities of 2.8 mg g^−1^ and 4.1 mg g^−^, respectively, without clogging, while mycorrhizal fungi introduced during planting enhance root uptake of PAHs and microplastics. Pilot monitoring at the Zhongshan Park catchment showed that effluent concentrations of microplastics dropped from 8400 particles L^−1^ at inflow to 1200 particles L^−1^ at outflow, with fibers and fragments retained primarily in the upper 10 cm of the biochar layer. Nanjing has extended the concept to river-edge “floating treatment wetlands” (FTWs) constructed from recycled PET frames planted with vetiver (*Chrysopogon zizanioides*) and water spinach (*Ipomoea aquatica*). Each 250 m^2^ FTW treats 4000 m^3^ d^−1^ of urban stormwater pumped directly from the Qinhuai River, achieving 55% removal of orthophosphate and 38% removal of Cu by combining plant assimilation with biofilm-mediated precipitation. Modular design allows units to be towed to hotspots after typhoon events, providing adaptive management that complements fixed green roofs and permeable pavements upstream. Life-cycle costing indicates that over a 20-year horizon, FTWs and bioswales together reduce combined sewer overflow volumes by 34% at one-third the capital and energy cost of expanding grey infrastructure while delivering co-benefits of urban heat island mitigation (mean surface temperature reduction 2.1 °C) and habitat provision for pollinators. Continued expansion of such green infrastructure is now embedded in the Yangtze River Protection Law, mandating that all new urban development’s >5 ha integrate runoff-control measures that achieve at least 80% annual pollutant-load reduction compared with pre-development baselines. Among these innovative approaches, satellite-borne hyperspectral remote sensing and IoT-based buoy networks represent technologies that are currently operational within the MEE monitoring framework (implemented stage). Cloud-based decision support platforms integrating multiple data streams are at the pilot and demonstration stage, with operational testing conducted in the Suzhou section of the Yangtze. Reinforcement-learning frameworks for 72 h pollutant trajectory forecasting remain at the early pilot stage, with initial validation completed but basin-wide deployment pending. Edge-computing buoys incorporating on-board surface-enhanced Raman spectroscopy for near-real-time emerging contaminant identification are currently at the prospective/development stage and require further technological maturation before field deployment.

Several critical implementation barriers threaten to delay the realization of the integrated management framework proposed in this review. First, the cost of basin-wide monitoring infrastructure expansion estimated at RMB 12–18 billion for comprehensive IoT buoy deployment and AI platform development exceeds current MEE budget allocations, requiring innovative financing mechanisms such as green bonds or public–private partnerships. Second, monitoring continuity is compromised by equipment maintenance challenges in remote upper basin reaches, where harsh winter conditions and limited road access result in average sensor downtime of 45–60 days per year. Third, data integration across 11 provincial jurisdictions is hindered by incompatible database architectures, inconsistent reporting formats, and inter-provincial competition that discourages data sharing. Fourth, inter-provincial coordination remains the most persistent institutional barrier: despite the YRPL’s mandate for joint enforcement, trans-boundary pollutant disputes between upstream and downstream provinces continue to arise, with resolution mechanisms that typically require 6–18 months of administrative negotiation. Addressing these barriers will require not only technological innovation but also institutional reform, capacity building, and sustained political commitment at the national level.

## 10. Conclusions

The Yangtze River Basin confronts deep-seated environmental challenges rooted in decades of rapid industrialization, yet recent policy measures have achieved notable progress in curbing point-source contamination. Persistent threats endure through legacy pollutants stored in sediments, diffuse agricultural runoff, and emerging contaminants such as microplastics, which collectively jeopardize both ecological stability and human health across the basin. Addressing these interconnected issues demands a holistic approach that merges targeted remediation technologies with nature-based solutions like phytoremediation and microbial treatment, all anchored within a robust governance framework. While biological and physical interventions show promise in restoring contaminated sites, their effectiveness depends on consistent enforcement of environmental regulations and cross-sectoral coordination. Moving forward, closing critical knowledge gaps, especially concerning long-term impacts of contaminant mixtures, through sustained research and basin-wide monitoring will be essential for evidence-based management. The path to a sustainable Yangtze requires a fundamental shift from reactive cleanup to proactive prevention, embedding circular economy principles that eliminate waste at its source and fostering inclusive collaboration among government, industry, and local communities. While the evidence base for point-source reduction and biological remediation is well established, the scalability of integrated green infrastructure approaches and the efficacy of AI-enhanced monitoring at the basin scale remain to be validated through long-term implementation studies. By integrating innovative solutions with strong policy commitments, the basin can be transformed from a symbol of environmental strain into a global exemplar of resilient, large-scale watershed governance, securing its vital resources for future generations while offering a replicable model for other mega-river systems worldwide.

## Figures and Tables

**Table 1:** Environmental contamination of the Yangtze River Delta and relevant sources causing soil, air, water, and sediment contamination.

Primary Contaminants	Sources	Type of Pollutants	Sample Collection Point/Area	Type of Sample	Reference Study
**Heavy metals**					
Lead (Pb)	Industrial activities, urban runoff, historical contamination	Pb	Yangtze River Delta, China	Soil	[76]
Cadmium (Cd)	Agricultural practices, industrial discharge, mining	Cd	Lower reaches of the Yangtze River, China	Soil	[77]
Mercury (Hg)	Industrial emissions, coal combustion, electronic waste	Hg	Yangtze River Delta, China	Soil	[49]
Chromium (Cr)	Industrial effluents, leather tanning, metal plating	Cr	Industrial regions across China	Soil	[78]
Arsenic (As)	Natural deposits, mining, pesticides	As	Yangtze River Delta, China	Groundwater	[41]
Copper (Cu)	Industrial discharge, mining, agricultural runoff	Cu	Yangtze River Delta, China	Soil	[79]
Zinc (Zn)	Industrial activities, galvanization, urban runoff	Zn	Yangtze River Delta, China	Soil	[43]
Nickel (Ni)	Industrial emissions, metal refining, batteries	Ni	Yangtze River Delta, China	Soil	[79]
Antimony (Sb)	Industrial activities, mining, vehicle brakes	Sb(III), Sb(V)	Yangtze River Delta, China	Soil	[80]
Fluoride (F)	Industrial discharge, natural geological sources	F	Informal landfills in the Yangtze River Delta, China	Groundwater	[18]
**Organic contaminants and Microplastics**					
Polycyclic Aromatic Hydrocarbons (PAHs)	Combustion of fossil fuels, industrial processes, vehicle emissions	PAHs (e.g., Benzo[a]pyrene)	Three Gorges Reservoir, China	Sediment	[64]
Organohalogenated Compounds	Industrial discharge, pesticide use, urban runoff	Chlorinated aliphatic hydrocarbons	Shallow groundwater in Shanghai, China	Groundwater	[81]
Pesticides (e.g., Atrazine)	Agricultural runoff, pesticide application	Atrazine	Yangtze River Delta, China	Soil	[65]
Hexachlorocyclohexane (HCH)	Historical pesticide use, industrial contamination	HCH, DDT	Agricultural soils across China	Soil	[82]
Microplastics	Urban runoff, wastewater discharge, plastic degradation	Various polymers	Yangtze River Estuary, China	Water, Sediment	[69]

**Table 2:** Summary of pollution indicator trajectories in the Yangtze Basin (2010–2022).

Indicator	Spatial Pattern	Temporal Trend	Primary Driver	Input Type	Policy Response
Dissolved Cd/Pb	Higher near industrial parks	Decline of 38–42% (2013–2022)	Point-source industrial controls	Legacy plus continuing	“Water Ten” plan; discharge permits
Hg (sediment flux)	Peaks at Poyang Lake, Three Gorges Reservoir	Decline of 68% (2007–2020)	Smelter closures; wet scrubbers	Primarily legacy	“Soil Ten” action; smelter phase-out
Σ_10_PAHs	Concentrated in Yangtze River Delta industrial zones	Persistent above 4000 ng/g (2020)	Industrial legacy; ongoing petrochemical activity	Legacy dominant	Partial controls; limited effectiveness
Microplastics	Highest in Shanghai estuary; storm-driven pulses	Increase of 295% (2013–2022); 9.4% per year	Plastic production growth; packaging waste	Continuing (new)	Minimal upstream reduction policies
Cr(VI) groundwater	Nanjing Qixia district plume	Stable or elevated up to 110 µg/L	Legacy electroplating	Legacy	Site remediation required
Zn (industrial)	Suzhou section hotspot	Decline of 55% (post-2019)	Permit system enforcement	Continuing	2019 Wastewater Discharge Permit

**Table 3:** Synthesis of ecological and human health risk evidence in the Yangtze River Basin.

Risk Domain	Evidence Type	Key Finding	Quantitative Value	Interpretation/Context
**Ecological**	Field observation	Benthic community impairment near industrial discharge	Species richness decline >50% within 5 km	Direct ecosystem impact; correlates with sediment Hg and PAH concentrations
	Field observation	Fish bioaccumulation in lower reaches	30% of predatory fish exceed 0.5 mg kg^−1^ Hg wet weight	Approaches 1.0 mg kg^−1^ consumption advisory threshold
	Field observation	Seasonal hypoxia downstream of Three Gorges Dam	Dissolved oxygen <2 mg L^−1^ during summer stratification	Recurring kill zones for bottom-dwelling species
	Laboratory study	Cadmium acute toxicity to zebrafish embryos	96 h LC_50_ = 125 µg L^−1^ in soft water	Upper basin tributary conditions; establishes acute threshold
	Laboratory study	Lead acute toxicity to zebrafish embryos	96 h LC_50_ = 890 µg L^−1^ in soft water	Lower toxicity than Cd; concentration–response reference
	Laboratory study	Microplastic sublethal effects on juvenile carp	10,000 particles L^−1^ induces feeding depression	Environmentally relevant concentration; behavioral endpoint
	Laboratory study	Oxidative stress from PAH-contaminated sediment	MDA levels increase 3× after 2-week exposure	Mechanistic pathway validation; biomarker response
	Laboratory study	Genotoxicity in carp erythrocytes	Significant DNA fragmentation at >500 ng g^−1^ B[a]P	Comet assay; sediment quality threshold indicator
	Modeled estimate	Cadmium species sensitivity distribution	HC_5_ = 1.2 mg kg^−1^ sediment (5% species affected)	40% of Yangtze Delta sediments exceed ecological threshold
	Modeled estimate	Mercury biomagnification	BMF = 3.8–6.2 per trophic level	Top predators accumulate highest burdens; dietary exposure driver
	Modeled estimate	Pesticide mixture risk in agricultural tributaries	65% of locations show probable risk to invertebrates	Combined organophosphate and pyrethroid exposures
**Human Health**	Epidemiological	Lung cancer mortality near petrochemical facilities	RR = 1.34 (males), 1.28 (females)	Significant excess mortality after smoking adjustment; 12,000-subject cohort
	Epidemiological	Childhood blood lead near former smelters	6.8 vs. 3.2 µg dL^−1^ (geometric mean)	15% of exposed children exceed 10 µg dL^−1^ intervention threshold
	Epidemiological	Preterm birth and chlorinated solvents	OR = 1.42 for TCE >5 µg L^−1^	Drinking water exposure in Shanghai suburbs
	Modeled estimate	Mercury intake via fish consumption	0.48 µg kg^−1^ bw day^−1^	84% of JECFA tolerable daily intake (0.57 µg kg^−1^ bw day^−1^)
	Modeled estimate	PAH cancer risk for recreational anglers	ILCR = 2.3 × 10^−4^	Exceeds 1.0 × 10^−4^ de minimis threshold; sediment contact scenario
	Modeled estimate	Microplastic drinking water risk	0.02 DALYs per capita annually	Below toxicological thresholds but includes uncertainty
	Biomonitoring	Breast milk PCB congener sum	180 ng g^−1^ lipid (median, 2020)	Decline from 340 ng g^−1^ (2005); comparable to global industrialized regions
	Biomonitoring	Hair mercury in fishing communities	1.9 µg g^−1^ geometric mean	22% exceed 2.0 µg g^−1^ reference for neurological symptoms
	Biomonitoring	Urinary 1-hydroxypyrene in urban populations	Elevated levels detected	Indicates combined inhalation and dietary PAH exposure
**Mixture Toxicity**	Laboratory	Cd-Pb synergistic toxicity to zebrafish	30–50% greater than additive	Concentration ratios typical of Yangtze sediments
	Laboratory	PAH mixture AHR activation	B[a]P + fluoranthene below individual thresholds	Receptor-mediated interaction; non-additive mechanism
	Laboratory	Microplastic pollutant carrier effect	1.5–3× increased effective toxicity	Hydrophobic organic pollutant delivery enhancement
	Field observation	Whole-sediment toxicity vs. individual contaminants	Observed > predicted from single-chemical models	Synergistic interactions; unmeasured organic toxicant contributions
	Modeled estimate	Cumulative hazard index for delta communities	HI = 2.4–4.7	Probable health concern; heavy metals + pesticides + solvents
	Modeled estimate	Dioxin-like TEQ in fish	12–45 pg g^−1^ lipid	Endocrine disruption risk range; RPF approach
	Modeled estimate	PAH mixture margin of exposure	Approaches threshold for developmental toxicity	Sensitive subpopulation consideration required

LC_50_ = median lethal concentration; MDA = malondialdehyde; HC_5_ = hazardous concentration for 5% of species; BMF = biomagnification factor; RR = relative risk; OR = odds ratio; TCE = trichloroethylene; bw = body weight; ILCR = incremental lifetime cancer risk; DALY = disability-adjusted life year; AHR = aryl hydrocarbon receptor; TEQ = toxic equivalency quotient; RPF = relative potency factor; HI = hazard index.

## Data Availability

No new data were created or analyzed in this study. Data sharing is not applicable to this article.
